# Phytochemicals from the Cocoa Shell Modulate Mitochondrial Function, Lipid and Glucose Metabolism in Hepatocytes via Activation of FGF21/ERK, AKT, and mTOR Pathways

**DOI:** 10.3390/antiox11010136

**Published:** 2022-01-08

**Authors:** Miguel Rebollo-Hernanz, Yolanda Aguilera, Maria A. Martin-Cabrejas, Elvira Gonzalez de Mejia

**Affiliations:** 1 Department of Agricultural Chemistry and Food Science, Universidad Autónoma de Madrid, 28049 Madrid, Spain; miguel.rebollo@uam.es (M.R.-H.); yolanda.aguilera@uam.es (Y.A.); maria.martin@uam.es (M.A.M.-C.); 2 Institute of Food Science Research, CIAL (UAM-CSIC), 28049 Madrid, Spain; 3 Department of Food Science and Human Nutrition, University of Illinois at Urbana-Champaign, Urbana, IL 61801, USA

**Keywords:** cocoa shell, cocoa by-products, antioxidants, theobromine, phenolic compounds, phytochemicals, non-alcoholic fatty liver disease, oxidative stress, mitochondrial function, metabolism

## Abstract

The cocoa shell is a by-product that may be revalorized as a source of bioactive compounds to prevent chronic cardiometabolic diseases. This study aimed to investigate the phytochemicals from the cocoa shell as targeted compounds for activating fibroblast growth factor 21 (FGF21) signaling and regulating non-alcoholic fatty liver disease (NAFLD)-related biomarkers linked to oxidative stress, mitochondrial function, and metabolism in hepatocytes. HepG2 cells treated with palmitic acid (PA, 500 µmol L^−1^) were used in an NAFLD cell model. Phytochemicals from the cocoa shell (50 µmol L^−1^) and an aqueous extract (CAE, 100 µg mL^−1^) enhanced ERK1/2 phosphorylation (1.7- to 3.3-fold) and FGF21 release (1.4- to 3.4-fold) via PPARα activation. Oxidative stress markers were reduced though Nrf-2 regulation. Mitochondrial function (mitochondrial respiration and ATP production) was protected by the PGC-1α pathway modulation. Cocoa shell phytochemicals reduced lipid accumulation (53–115%) and fatty acid synthase activity (59–93%) and prompted CPT-1 activity. Glucose uptake and glucokinase activity were enhanced, whereas glucose production and phosphoenolpyruvate carboxykinase activity were diminished. The increase in the phosphorylation of the insulin receptor, AKT, AMPKα, mTOR, and ERK1/2 conduced to the regulation of hepatic mitochondrial function and energy metabolism. For the first time, the cocoa shell phytochemicals are proved to modulate FGF21 signaling. Results demonstrate the in vitro preventive effect of the phytochemicals from the cocoa shell on NAFLD.

## 1. Introduction

Chronic diseases account for 71% of all fatalities globally, according to the World Health Organization [1]. Dysfunctional metabolic processes in cells produce energy and redox imbalances in the body, leading to various pathophysiological metabolic conditions. Obesity, insulin resistance, type 2 diabetes, hypertension, hyperlipidemia, and metabolic syndrome are the most common chronic metabolic diseases [2]. These conditions involve the development of non-alcoholic fatty liver disease (NAFLD), a disorder characterized by excessive fat deposition in the form of triglycerides in the liver (steatosis) [3]. The current worldwide prevalence of NAFLD is estimated to be 24% [4]. These diseases could be prevented through nutrition and appropriate dietary habits [5].

Fibroblast growth factor 21 (FGF21) is a hormone that controls critical metabolic processes [6]. Due to its positive effects on lipid and glucose metabolism, FGF21 has emerged as a promising therapeutic target for metabolic diseases. Even though most research studies reveal that FGF21 treatment has positive metabolic effects [7], FGF21 serum levels are paradoxically elevated under obese and diabetic conditions, which has generated the concept of FGF21 resistance [8]. The primary source of circulating FGF21 is the liver, where FGF21 production is regulated by the peroxisome proliferator-activated receptor α (PPARα) [9]. FGF21 active binding with FGF receptor (FGFR) and interaction with a coreceptor, β-klotho (β-KL), is required for signal transduction. FGF21 interacts with FGFR with extremely low affinity [10]. The metabolic action of FGF21 is governed through the mitogen-activated protein kinase (MAPK) signaling cascade. When FGF21 binds to FGFR/β-KL, extracellular signal-regulated kinase (ERK) 1/2 is rapidly phosphorylated. Active p-ERK1/2 is then translocated to the nucleus, activating downstream pathways, consequently controlling biological processes [11]. In the liver, FGF21 positively controls the phosphoinositide 3-kinase (PI3K)/protein kinase B (AKT), insulin-like growth factor 1 (IGF-1), and mammalian target of rapamycin (mTOR) pathways. In addition, it increases fat utilization and energy expenditure, stimulates fatty acid oxidation, lowers hepatic triglyceride levels, and enhances glucose tolerance and insulin sensitivity. FGF21 may also protect from NAFLD lipotoxicity, leading to mitochondrial dysfunction, reactive oxygen species (ROS) generation, and inflammation [12].

Cocoa shell is the major by-product of cocoa processing (12–20% of cocoa seed) removed from the bean during the roasting process, produced at a rate of roughly 700 k tons per year, and formerly considered a waste [13]. Our previous studies demonstrated the applicability of phytochemicals extracted from cocoa shells on the modulation of inflammation, insulin resistance, and mitochondrial function in macrophages and adipocytes [14,15]. Cocoa shell phytochemicals reduced LPS-triggered inflammatory factors secretion (TNF-α, MCP-1, NO, and PGE-2) and stimulated adipocyte insulin sensitivity by prompting GLUT-4 translocation and glucose uptake via insulin/PI3K-Akt signaling activation. Likewise, the phytochemicals from the cocoa shell stimulated mitochondrial respiration and ATP production and reduced oxidative stress [14,15]. The use of cocoa by-products as a source of biologically active phytochemicals can be considered a sustainable strategy for health promotion or disease prevention.

Limited research has shown the effects of dietary bioactive compounds on the modulation of FGF21 secretion from the liver or the activation of its signaling pathway. The literature has only evidenced the effects of long-chain fatty acids (enhancing FRFR/β-KL expression) [16], betaine (raising FGF21 secretion) [17], and curcumin (favoring both FRFR/β-KL and FGF21 expression) [18]. Likewise, there are no studies on the impact of the phytochemicals from cocoa shells on liver function under metabolic syndrome conditions. We hypothesized that the phytochemicals in the aqueous extract from cocoa shells would activate FGF21 signaling in hepatocytes, thereby protecting the liver from metabolic stress. Hence, this work aimed to evaluate the potential of pure phytochemicals and an extract from the cocoa shell to activate the hepatic FGF21 signaling, and the subsequent modulation of metabolic syndrome-related biomarkers associated with oxidative stress, mitochondrial function, and lipid and glucose metabolism, in hepatocytes in vitro.

## 2. Materials and Methods

### 2.1. Materials

Minimum essential medium (MEM) and sodium pyruvate were purchased from Corning Cellgro (Manassas, VA, USA). Fetal bovine serum (FBS), penicillin–streptomycin (100×), and 0.25% trypsin-EDTA were obtained from Gibco Life Technologies (Grand Island, NY, USA). Pure phytochemicals (purity ≥ 96%) including theobromine (TH), protocatechuic acid (PCA), procyanidin B2 (PB2), epicatechin (EPI), and catechin (CAT) were purchased from Sigma-Aldrich (St. Louis, MO, USA) and Extrasynthese (Genay, France). Recombinant human FGF21 and PD173074 were obtained from R&D Systems (Minneapolis, MN, USA) and AdoQ Bioscience (Irvine, CA, USA), respectively.

### 2.2. Cocoa Shell Aqueous Extract (CAE) Preparation and Phytochemical Characterization by UPLC–MS/MS

Chocolates Santocildes (Castrocontrigo, León, Spain) provided the cocoa shell. A phenolic-rich aqueous extract from the cocoa shell (CAE) was produced using previously published extraction methods [19]. After milling and sieving, the ground cocoa shell (10 g) was added to 500 mL of boiling water (100 °C) and stirred for 90 min. The extract containing the aqueous soluble components from the cocoa shell was freeze-dried and kept at −20 °C until further usage.

The targeted phytochemicals were analyzed using UPLC–ESI–MS/MS using a previously established methodology [20]. After filtering (0.22 µm), an internal standard 4-hydroxybenzoic-2,3,5,6-d4 acid solution (Sigma-Aldrich, St Louis, MO, USA) was added to the samples in a 1:5 (*v*/*v*) proportion. The column utilized was a Waters BEH-C18 with 2.1 × 100 mm and 1.7 µm particle sizes. The liquid chromatographic system employed a Waters Acquity UPLC (Milford, MA, USA) with a binary pump, a heated autosampler (10 °C), and a heated column compartment (40 °C). The LC effluent was injected into an Acquity TQD tandem quadrupole mass spectrometer with a Z-spray ESI source. A gradient composed of solvents A (water:acetic acid, 98:2 *v*/*v*) and B (acetonitrile:acetic acid, 98:2 *v*/*v*) was applied at a flow rate of 0.5 mL min^−1^ as follows: 0 min, 0.1% B; 1.5 min, 0.1% B; 11.17 min, 16.3% B; 11.5 min, 18.4% B; 14 min, 18.4% B; 14.1 min, 99.9% B; 15.5 min, 99.9% B; 15.6 min, 0.1% B; and 18 min, 0.1% B. The injection volume was 2 μL. The MRM mode was used to record the transition of the parent and product ions for each compound. The ESI was utilized in negative ionization mode for phenolics and positive ionization mode for methylxanthines and anthocyanins (which were not identified). The ESI parameters were as follows: capillary voltage, 3 kV; source temperature, 130 °C; desolvation temperature, 400 °C; desolvation gas (N_2_) flow rate, 750 L h^−1^; cone gas (N_2_) flow rate, 60 L h^−1^. All chemicals were quantified using their own standard calibration curves.

### 2.3. Cell Culture

The HepG2 human hepatocyte cell line was acquired from the American Type Culture Collection (Manassas, VA, USA) and cultured at 37 °C in a 5% CO_2_ environment. HepG2 cells were grown in minimum essential medium (MEM) supplemented with 10% fetal bovine serum (FBS), 1% penicillin–streptomycin, and 1% sodium pyruvate. The cells were seeded in a flask at a density of 5 × 10^5^ cells cm^−2^.

### 2.4. Experimental Design

Pure phytochemicals from the cocoa shell (TH, PCA, PB2, EPI, and CAT; 5–100 µmol L^−1^), CAE (10–500 µg mL^−1^), or recombinant human FGF21 (5–50 nmol L^−1^) were applied to hepatocytes for 24 h in the presence or absence of palmitic acid (PA) (500 µmol L^−1^). Supernatants were collected and kept at −80 °C until further analysis. Cells were washed twice with ice-cold PBS before being lysed with the RIPA Lysis Buffer System (Santa Cruz Biotechnology, CA, USA) and centrifuged at 10,000× *g* for 10 min at 4 °C to remove cell debris before being kept at −80 °C until further analysis. The protein concentration in cell lysates was quantified using bovine serum albumin (BSA) as standard with the DC protein assay (BioRad, Richmond, CA, USA).

### 2.5. Cell Viability

Cell viability of cells treated for 24 h with the cocoa shell pure phytochemicals (5–100 µmol L^−1^), CAE (10–500 µg mL^−1^), or recombinant human FGF21 (5–50 nmol L^−1^) in the presence or absence of palmitic acid (PA) (500 µmol L^−1^) was measured using the CellTiter 96 Aqueous One Solution Proliferation assay (Promega Corporation, Madison, WI, USA).

### 2.6. Assessment of the Effect of the Cocoa Shell Phytochemicals on FGF21 Signaling Activation

#### 2.6.1. In Silico Molecular Docking of the Interaction of Phytochemicals with the FGF21 Receptor

The studied phytochemicals from the cocoa shell (TH, PCA, PB2, EPI, and CAT) were tested as possible ligands for the different subunits of the FGF21 receptor using molecular docking (FGFR1: 5A4C; β-klotho: 5VAQ). The Protein Data Bank (PBD) website (http://www.rcsb.org/pdb/home/home.do, accessed on 20 September 2021) was used to obtain their 3D crystal structures. The binding sites of co-crystallized inhibitors or substrates were chosen as the docking area’s center. The ligands (TH, PCA, PB2, EPI, and CAT) structures were acquired from the PubChem Compound database (https://pubchem.ncbi.nlm.nih.gov/pubchem, accessed on 20 September 2021). AutoDock Tools was used to open ligand files to apply Gasteiger partial charges and determine the root of each structure set’s rotatable bonds. AutoDock Tools was used to set the search space dimensions, center point, and flexible torsions, and AutoDock Vina was used to perform the docking calculations. The different phytochemicals were docked to the FGF21-binding site in FGFR1 (*x*, 90.58; *y*, 0.15; *z*, 14.27) and β-klotho (*x*, 54.53; *y*, 35.60; *z*, 52.79). One hundred runs were performed for each ligand, and the conformation with the highest binding affinity (lowest binding energy, BE) was recorded. PRODIGY was used to calculate protein–ligand BE [21] and protein–protein (FGF21) BE for the co-crystallized complexes retrieved from PBD [22]. Discovery Studio 2017 R2 Client (Dassault Systèmes Biovia Corp, San Diego, CA, USA) was used to view protein–ligand interactions and binding modes.

#### 2.6.2. ERK1/2 Phosphorylation Evaluation

The activation of the FGFR/KBL complex and the phosphorylation of ERK1/2 were identified as indicators of FGF21 sensitivity. The phosphorylation of ERK1/2 was measured in HepG2 cell lysates after treatment with pure phytochemicals from the cocoa shell (50 µmol L^−1^), CAE (100 µg mL^−1^), or recombinant human FGF21 (20 nmol L^−1^) for 24 h in the presence or absence of palmitic acid (PA) (500 µmol L^−1^) or PD173074 (50 nmol L^−1^) using commercial ELISA kits according to the manufacturer’s instructions (R&D Systems, Minneapolis, MN, USA). PD173074 was used as a selective FGFR inhibitor to evaluate the specificity of treatments on enhancing ERK1/2 phosphorylation via FGFR signaling.

#### 2.6.3. FGF21 Release Quantification

The secretion of FGF21 to the HepG2 culture media was measured according to the manufacturer’s instructions using commercial ELISA kits (R&D Systems, Minneapolis, MN, USA).

### 2.7. Evaluation of the Effect of the Phytochemicals from the Cocoa Shell on Hepatic Lipotoxicity

#### 2.7.1. Lactate Dehydrogenase (LDH) Cytotoxicity Assay

The LDH activity was quantified in cell culture supernatants as an indicator of lipotoxicity or cell damage using the Pierce LDH cytotoxicity assay kit (Thermo Scientific, Rockford, IL, USA).

#### 2.7.2. Quantification of Cytokines Release

TNF-α, IL-6, and IL-1β were measured in cell supernatants using ELISA commercial kits of each cytokine (R&D Technologies, Minneapolis, MN, USA) according to the manufacturer’s instructions.

#### 2.7.3. Determination of Nitric Oxide Synthase (NOS) Activity

NOS activity was assayed in cell lysates using a total nitric oxide synthase activity kit (BioVision, Milpitas, CA, USA) following the manufacturer’s instructions. The NOS activity was expressed as mU mg^−1^ protein.

### 2.8. Evaluation of the Effect of the Phytochemicals from the Cocoa Shell on Oxidative Stress and Mitochondrial Function

#### 2.8.1. Detection of Intracellular ROS, Mitochondrial Superoxide, Mitochondrial Membrane Potential (ΔΨm), and Enzymatic Antioxidant Activity

ROS generation was assessed following the 24 h treatment with palmitic acid (500 μmol L^−1^) and pure phytochemicals (50 μmol L^−1^), CAE (100 μg mL^−1^), or FGF21 (20 nmol L^−1^). The cells were cultured for 1 h in MEM with 2′,7′dichlorodihydrofluorescein diacetate (DCFDA, 25 μmol L^−1^). The cells were then rinsed with PBS, and the fluorescence was measured at excitation/emission wavelengths of 485 nm/535 nm, respectively. Mitochondrial superoxide was measured by treating cells with 50 μmol L^−1^ Mitosox Red (Invitrogen Molecular Probes, Carlsbad, CA, USA) and detecting fluorescence at 510 nm excitation and 580 nm emission wavelengths. The mitochondrial membrane potential (ΔΨm) was measured using the mitochondria-specific fluorescent dye, JC1 (Thermo Fisher, Skokie, IL, USA), following the manufacturer’s instructions. JC1 aggregates were identified at 550/590 nm (excitation/emission), while JC1 monomers were found at 485/535 nm (excitation/emission). The JC1 aggregates/monomers ratio was determined for each condition as an indication of mitochondrial functioning.

NADPH oxidase activity was measured as previously described following 24 h treatment with palmitic acid (500 μmol L^−1^) and pure phytochemicals (50 μmol L^−1^), CAE (100 μg mL^−1^), or FGF21 (20 nmol L^−1^) [23]. HepG2 cells were treated with 250 μmol L^−1^ NADPH for 5 min, and NADPH consumption was measured by the change in the absorbance at 340 nm.

Cell lysates were used for measuring superoxide dismutase (SOD) and catalase activities. For the SOD activity, the reaction was initiated by adding nitro-blue tetrazolium (60 μmol L^−1^) in a final volume of 250 μL [24]. The rise in absorbance (570 nm) was measured every 60 s for 15 min at 37 °C. Similarly, the breakdown of hydrogen peroxide was used to evaluate catalase activity [25]. The reaction was initiated by adding 30 mmol L^−1^ H_2_O_2_ into the mixture of cell lysate and buffer (100 mmol L^−1^ PBS, pH 6.8). Changes in absorbance measured at 240 nm (each 5 s for 5 min, at 37 °C) were used to measure catalase activity.

#### 2.8.2. Assessment of Mitochondrial Content and Function

After the 24 h treatment with palmitic acid (500 μmol L^−1^) and pure phytochemicals (50 μmol L^−1^), CAE (100 μg mL^−1^), or FGF21 (20 nmol L^−1^), mitochondrial mass was measured using Mitotracker Green (Mitotracker Deep Green FM, Invitrogen). The fluorescence intensity was measured at excitation and emission wavelengths of 644 nm and 665 nm, respectively. The oxygen consumption rate (OCR), as a direct indicator of mitochondrial activity, was measured using a kit guided by the manufacturer indications (ab197243; Abcam, Cambridge, UK). Citrate synthase (CS) activity was measured using a kit (Cayman Chemical Item No. 701040, Ann Arbor, MI, USA) according to the manufacturer’s instructions. OXPHOS complex I (CI) activity was detected using a previously described method [26]. In summary, HepG2 hepatocytes were cultured and lysed as previously indicated. Then, the mitochondrial fraction was separated using the mitochondria isolation kit (Thermo Scientific, Rockford, IL, USA). The NADH oxidation rate was used to measure CI activity in the isolated mitochondrial fraction. Briefly, mitochondrial protein fractions were mixed with KH_2_PO_4_ buffer (50 mmol L^−1^, pH 7.5) containing BSA (3.75 mg mL^−1^), decylubiquinone (100 μmol L^−1^), and NADH (100 μmol L^−1^). An ATP detection assay kit (Cayman) was used to quantify the ATP concentration in cell lysates according to the manufacturer’s instructions (Cayman Chemical, No. 700410).

### 2.9. Evaluation of the Effect of the Phytochemicals from the Cocoa Shell on Lipid Metabolism

#### 2.9.1. Determination of Cellular Lipid Accumulation

Lipid staining with Oil Red O was carried out as previously reported [27]. Pure phytochemicals (50 µmol L^−1^), CAE (100 µg mL^−1^), or FGF21 (20 nmol L^−1^) were added to HepG2 cells grown in 24-well plates for 24 h. Before normalization with cell viability, results were expressed as a fold change in lipid accumulation compared to the non-treated control.

#### 2.9.2. Assessment of Lipolysis

The culture media was collected from palmitic acid-treated HepG2 cells after the 24 h co-treatment with palmitic acid (500 µmol L^−1^), pure phytochemicals (50 µmol L^−1^), CAE (100 µg mL^−1^), or FGF21 (20 nmol L^−1^) and tested for glycerol quantification using a glycerol cell-based assay kit (Cayman Chemical Item No. 10011725). The cell lysates were assayed for intracellular triglycerides (TAG) and lipase activity using commercial kits (Cayman Chemical Item No. 10010303 and 700640, respectively).

#### 2.9.3. Measurement of Fatty Acid Synthase (FASN) Activity

FASN activity was measured by assessing the rate of NADPH oxidation, as previously described [28]. Cell lysates were mixed with EDTA (1 mmol L^−1^), dithiothreitol (1 mmol L^−1^), acetyl-CoA (30 μmol L^−1^), and NADPH (0.15 mmol L^−1^) in a final volume of 300 µL. To evaluate background NADPH oxidation, the basal reaction was recorded at 340 nm for 3 min. Then, the fatty acid synthesis reaction was started by adding malonyl-CoA (50 mol L^−1^), and the reduction in absorbance at 340 nm was measured for 20 min. The FASN activity was expressed in nmol min^−1^ mg^−1^.

#### 2.9.4. Measurement of Carnitine Palmitoyltransferase 1 (CPT-1) Activity

CPT-1 activity was measured as previously described [29,30]. Cell lysates CPT-1 activity was assayed in 200 μL reaction buffer containing Tris-HCl (100 mmol L^−1^, pH 7.4), DTNB (0.12 mmol L^−1^), BSA (20 μmol L^−1^), TritonX-100 (0.09%), and palmitoyl-CoA (75 μmol L^−1^). The reaction was initiated by adding L-carnitine (75 μmol L^−1^), and the absorbance was recorded at 412 nm after incubating 3 min at 37 °C. The CPT-1 activity was expressed in nmol min^−1^ mg^−1^.

### 2.10. Evaluation of the Effect of the Phytochemicals from the Cocoa Shell on Glucose Metabolism

#### 2.10.1. Determination of Glucose Uptake

The impact of phytochemicals from the cocoa shell on glucose uptake was evaluated using 2-deoxy-2-[(7-nitro-2,1,3-benzoxadiazol-4-yl)amino]-D-glucose (2-NBDG) uptake as previously described [28]. HepG2 cells were grown in a black 96-well plate with a transparent bottom and exposed to palmitic acid (500 μmol L^−1^) and pure phytochemicals (50 μmol L^−1^), CAE (100 μg mL^−1^), or FGF21 (20 nmol L^−1^). After the 24 h treatment, the cells were incubated for 2 h in glucose-free MEM containing 100 μmol L^−1^ 2-NBDG. After washing the cells with PBS, the fluorescence was measured at 485 nm/535 nm excitation/emission wavelengths.

#### 2.10.2. Measurement of Glucokinase (GK) Activity

GK activity was measured using a previously reported method [31]. A solution of glucose-6 phosphate dehydrogenase (100 U mL^−1^) was added to a reaction cocktail comprising glucose, Tris-HCl buffer (pH 9), MgCl_2_, ATP, and NADP. After 5 min of equilibration, the cell lysates were added, and the absorbance was measured at 320 nm for 10 min at 37 °C. Hexokinase activity was corrected by subtracting the activity measured with 0.5 mmol L^−1^ glucose (which only measures low *Km* hexokinases) from the activity recorded with 360 mmol L^−1^ glucose (measures all hexokinases, including GK).

#### 2.10.3. Assessment of Gluconeogenesis

HepG2 cells were seeded in 24-well plates and treated for 24 h with palmitic acid (500 μmol L^−1^). The culture mediums were supplemented with pure phytochemicals (50 μmol L^−1^), CAE (100 μg mL^−1^), or FGF21 (20 nmol L^−1^). The HepG2 culture medium was then switched to glucose production buffer (glucose-free MEM supplemented with 20 mmol L^−1^ sodium lactate and 2 mmol L^−1^ sodium pyruvate) and incubated for 4 h. Following that, D-glucose was detected in the medium using an Amplex Red Glucose/Glucose Oxidase Assay Kit (Invitrogen, Carlsbad, CA, USA) according to the manufacturer’s instructions.

#### 2.10.4. Measurement of Phosphoenolpyruvate Carboxykinase (PEPCK) Activity

PEPCK was determined by the carboxylation of phosphoenolpyruvate to yield oxaloacetic acid in the presence of NADH, as previously reported [32]. Cell lysates were added to the reaction buffer (50 mmol L^−1^ Tris–HCl, 2 mmol L^−1^ MnCl_2_, 1.25 mmol L^−1^ inosine diphosphate, 50 mmol L^−1^ of KHCO_3_, 2.5 units mL^−1^ malate dehydrogenase, and 0.15 mmol L^−1^ NADH) in a 96-well plate. The reaction was initiated by adding 10 μL of 0.4 mol L^−1^ phosphoenolpyruvate, and the process was terminated after 10 min of incubation at 37 °C by adding 6 mmol L^−1^ HCl by placing the tube on ice. The absorbance of the final mixture was measured at 340 nm.

### 2.11. Evaluation of Protein Expression and Phosphorylation by Western Blot

The impact of the cocoa shell phytochemicals of the protein expression levels PPARα, p-Nrf2/Nrf2, p-AMPK^T172^/AMPK, PGC-1α, OXPHOS proteins, p-Akt1^S473^/Akt1, and GLUT-2 was evaluated by Western blotting. HepG2 cells co-treated with palmitic acid (500 µmol L^−1^), pure phytochemicals (50 µmol L^−1^), CAE (100 µg mL^−1^), or FGF21 (20 nmol L^−1^) were rinsed with precooled PBS, lysed in RIPA buffer, and centrifuged (10,000× *g*, 4 °C, 10 min) to remove cell debris. A similar amount of cell lysate protein (20 μg per lane) was subjected to 4–20% gradient SDS–polyacrylamide (SDS–PAGE) gels and transferred onto PVDF membranes. The membranes were blocked with 5% (*w*/*v*) nonfat dry milk in 0.1% Tris-buffered saline Tween 20 (1 h, RT) and probed with primary antibodies overnight at 4 °C. The membranes were then incubated with horseradish peroxidase-conjugated secondary antibody (1:5000, 1 h, RT; GE Healthcare, Buckinghamshire, UK). Immunoreactive bands were visualized using the ECL Prime Western Blotting kit (GE Healthcare, Buckinghamshire, UK), and images were acquired on an ImageQuant 800 System (GE Healthcare, Buckinghamshire, UK). To detect OXPHOS protein expression level, mitochondrial fractions were obtained from cell lysates using a mitochondria isolation kit (Thermo ScientificTM, Rockford, IL, USA). An equal amount of mitochondria protein (5 μg per lane) was separated by electrophoresis and transferred onto PVDF membranes, which were afterwards probed with MitoProfile^®^ Total OXPHOS WB (MitoProfile, Eugene, OR, USA) antibody cocktail. Protein expression levels were calculated relative to an appropriate loading control (β-actin for whole cell lysate and VDAC1 for mitochondrial fraction).

### 2.12. Evaluation of the Effect of the Cocoa Shell Phytochemicals on Metabolic Related Signaling Pathways Phosphorylation Pattern

HepG2 hepatocytes were cultured and treated with palmitic acid (500 µmol L^−1^) in the presence/absence of CAE (100 µg mL^−1^) for 24 h. After treatment, the cells were serum-starved for 30 min, followed by a 10 min stimulation with 10 ng mL^−1^ of insulin. Cell lysates were applied following the manufacturer’s instructions (AAH-INSR and AAH-AKT, RayBiotech, Peachtree Corners, GA, USA). Array signals for 27 different protein phosphorylation sites were visualized on a GelLogic 4000 Pro Imaging System (Carestream Health, Inc., Rochester, NY, USA), and phosphorylation levels were normalized for protein content.

### 2.13. Bioinformatic Analysis

The resulting differentially phosphorylated proteins and protein–protein interactions were searched using Metascape [33]. The differentially expressed proteins were categorized based on the biological process and molecular function and further analyzed for Kyoto Encyclopedia of Genes and Genomes (KEGG) pathway enrichment analysis using the KEGG database [34]. The protein–protein interacting network and the enrichment analysis of the studied proteins and their nearest functional and predicted associations were established.

### 2.14. Statistical Analysis

Experiments were carried out in triplicate. The results were reported as the mean ± standard deviation (SD) (*n* = 3) and analyzed using the *T*-test or one-way ANOVA and the post hoc Tukey test. Differences were considered significant at *p* < 0.05. SPSS 24.0 was used for the statistical analysis of the results.

## 3. Results and Discussion

### 3.1. Theobromine and Protocatechuic Acid Are the Major Compounds in the Cocoa Shell Extract

**Figure 1:**
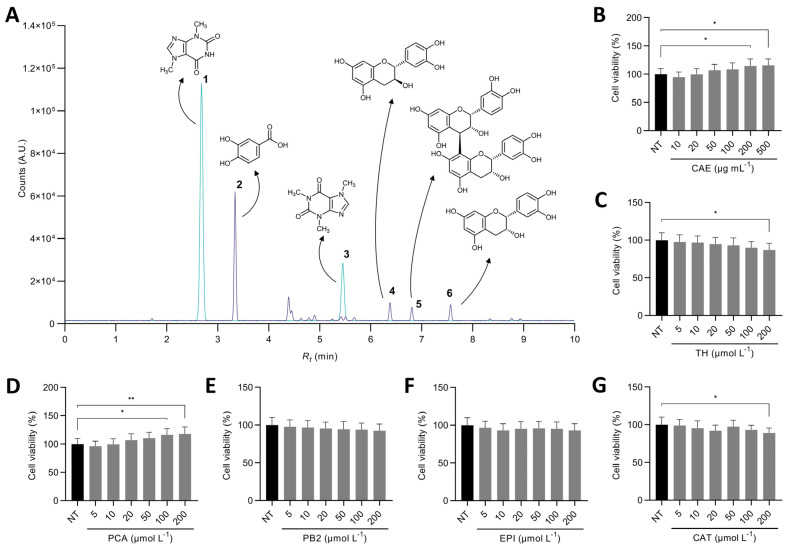
Superimposed UPLC–MS/MS chromatograms of the targeted analysis of phytochemicals from the cocoa shell aqueous extract (CAE) operating the MS detector in negative and positive modes (**A**). Primary compounds found in CAE were theobromine (1), protocatechuic acid (2), caffeine (3), catechin (4), procyanidin B2 (5), and epicatechin (6). Cell viability of HepG2 cells treated with CAE (**B**), theobromine (TH) (**C**), protocatechuic acid (PCA) (**D**), procyanidin B2 (PB2) (**E**), (−)-epicatechin (EPI) (**F**), and (+)-catechin (**G**). The results are expressed as mean ± SD (*n* = 3). Asterisks (* or **) denote significant differences (*p* < 0.05 or *p* < 0.01, respectively) according to the *t*-test between the non-treated control (NT) and each of the concentrations of CAE or pure compounds.

A comprehensive UPLC–MS/MS analysis of the phytochemical profile of the CAE showed that the cocoa shell was primarily composed of theobromine (10.03 mg g^−1^), corresponding to 80.5% of the compounds detected in the MS positive mode (Figure 1A, Supplementary Table S1). Protocatechuic acid was the main phenolic compound found (0.76 mg g^−1^), followed by flavanols, both monomers ((+)-catechin (0.20 mg g^−1^) and (−)-epicatechin (0.22 mg g^−1^) and dimers (procyanidin B2, 0.22 mg g^−1^) (Supplementary Table S1). Together, these phenolics accounted for 83.0% of compounds detected in the MS negative mode. Multiple extraction techniques (pressurized solvents, microwave-assisted extraction, maceration) have been investigated to separate the high-value phytochemicals from the cocoa shell. The concentration of obtained phytochemicals varied among studies because of the technique used per se and the intrinsic differences among cocoa shell samples. Nevertheless, regardless of the study, high theobromine and flavanols extracts have been obtained [35,36,37,38]. Therefore, these phytochemicals were considered for cell culture studies to investigate their properties in preventing NAFLD. Treating HepG2 cells with CAE (10–500 µg mL^−1^) or the phytochemicals from the cocoa shell in the form of pure compounds (5–200 µmol L^−1^) did not cause cell cytotoxicity at the concentrations tested (*p* > 0.05) (Figure 1B–G). Based on the absence of cytotoxicity and the effects observed in previous reports, the concentrations for the following experiments were selected (CAE 100 µg mL^−1^, and pure phytochemicals 50 µmol L^−1^) [15,39,40]. For preliminary experiments, we evaluated the effects of these compounds on ROS production and mitochondrial content (Supplementary Figure S1). Non-significant effects (*p* > 0.05) were observed, so the following experiments were carried out under PA-stimulatory conditions.

### 3.2. Protocatechuic Acid and Procyanidin B2 Mimicked FGF21 and Stimulated ERK Signaling

**Figure 2:**
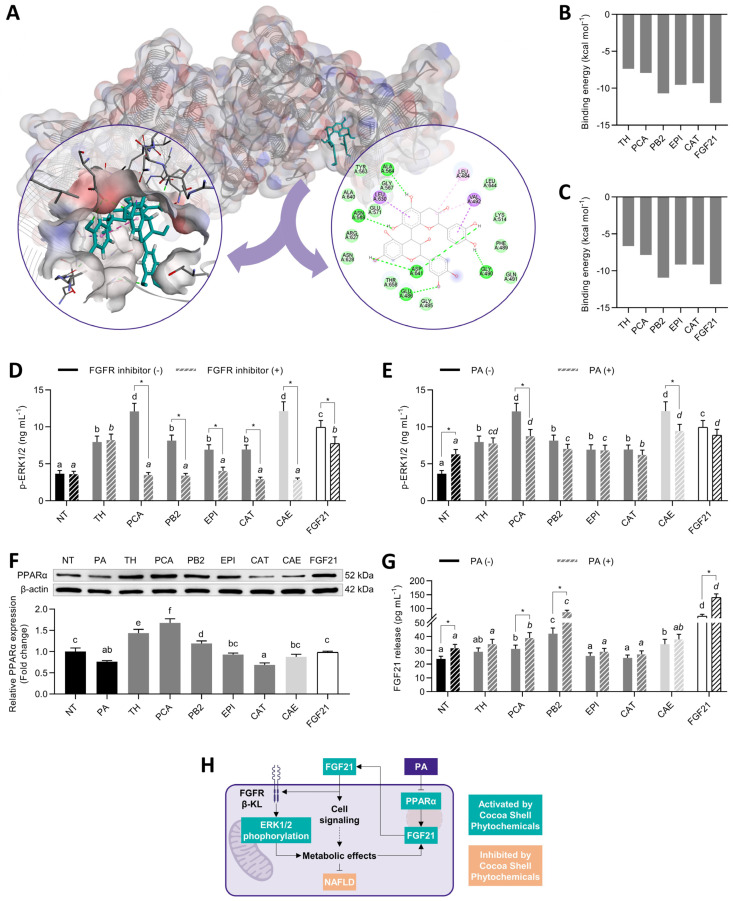
Activating effects of pure phytochemicals from the cocoa shell (50 µmol L^−1^), aqueous extract (CAE, 100 µg mL^−1^), and FGF21 (20 nmol L^−1^), in the presence of PD173074 (FGFR1 inhibitor, 50 nmol L^−1^) or palmitic acid (PA, 500 µmol L^−1^), on FGF21 signaling in HepG2 human hepatocytes. Phytochemicals interacted in silico with the subunits of the FGF21R (**A**), exhibiting different binding energies for FGFR1 (**B**) and β-klotho (**C**), leading to increases in ERK1/2 phosphorylation (**D**,**E**). Simultaneously, HepG2 exhibited increased PPARα protein expression (**F**) and FGF21 release (**G**). Integrative diagram illustrating the effects of the phytochemicals from the cocoa shell on FGF21 signaling activation (**H**). The results are expressed as mean ± SD (*n* = 3). Bars with different letters (a–f) significantly (*p* < 0.05) differ according to ANOVA and Tukey’s multiple range test. Non-italicized letters indicate differences in the absence of FGFR inhibitor or PA, whereas italicized letters indicate differences in the presence of the FGFR inhibitor or PA. Asterisks (*) denote significant differences (*p* < 0.05) according to the *t*-test between two experimental conditions of the same treatment. NT: non-treated cells; TH: theobromine; PCA: protocatechuic acid; PB2: procyanidin B2; EPI: epicatechin; CAT: catechin; FGF21: fibroblast growth factor 21; β-KL: β-klotho.

The phytochemicals from the cocoa shell and FGF21 were docked in silico with FRFR1 and β-klotho to elucidate the potential mechanism of action of the molecules associated with receptor activation [41]. All phytochemicals exhibited strong interactions with both FGFR1 and β-klotho. Figure 2A depicts the lowest energy poses for the interaction FGFR1–PB2. This compound exhibited the lowest binding energy (−10.7 kcal mol^−1^) comparable to FGF21 itself (−12.0 kcal mol^−1^). The other biomolecules ranged from −7.4 to −9.6 kcal mol^−1^ (Figure 2B). The strong interaction with the FGF21 activation loop (Ala564, Asp641) through hydrogen bonds, π–cation, and π–anion (strong interactions) was mainly responsible for the complexes’ stabilization, notwithstanding the contribution of hydrophobic and Van der Waals interactions. Comparably, PB2 interacted with β-klotho (−10.9 kcal mol^−1^) to a similar extent as FGF21 (−11.8 kcal mol^−1^), and the other phytochemicals exhibited modest interactions (from −6.6 to −9.1 kcal mol^−1^) (Figure 2C).

This interaction with the ligand binding pocket in the glycoside hydrolase-like domain was also stabilized by hydrogen bonds and π–anion interactions with Glu693, a conserved residue that interacts with the Ser–Pro–Ser motif of the FGF21 C-terminal tail [42]. As a result of the interaction with FGF21, hepatocytes exhibited increases in ERK1/2 phosphorylation (Figure 2D). PCA and CAE enhanced 3.3-fold p-ERK1/2, whereas FGF21, 2.7-fold, and the other treatments between 2.2- and 1.9-fold (*p* < 0.05). An FGFR1 inhibitor (PD173074) was added as a co-treatment to verify that ERK1/2 phosphorylation was FGFR1-dependent. Hence, p-ERK1/2 decayed from 21 to 76% (*p* < 0.05), but in the TH treatment. Considering that saturated fatty acids could impair FGF21 signaling [43], p-ERK1/2 values were also evaluated in the presence of palmitic acid (PA) (Figure 2E). We used HepG2 hepatocytes treated with 500 µmol L^−1^ PA to mimic the conditions established in the liver under NAFLD states. PA-treated cells showed 1.7-fold ERK1/2 phosphorylation compared with NT cells. However, no changes were observed except in PCA and CAE treatments, which repressed the phosphorylation by 28 and 22% (*p* < 0.05), respectively. This study is the first report investigating food phytochemicals on FGF21 signaling activation to the extent of our knowledge. FGF21-receptor agonists are recently emerging as active agents for preventing or reducing type 2 diabetes and NAFLD [44].

Since FGF21 can act as an autocrine molecule, another way to activate FGF21 signaling is by promoting FGF21 release. In both basal and NAFLD conditions (Figure 2F), phytochemicals enhanced PPARα expression (1.2- to 2.2-fold, *p* < 0.05), except for CAT. However, only PCA, PB2, and CAE elevated (*p* < 0.05) FGF21 secretion (31%, 77%, and 45%, respectively, in basal conditions; 23%, 173%, and 20%, respectively, in PA-stimulated hepatocytes) (Figure 2G). FGF21 stimulated its expression in a cyclic manner (Figure 2H). FGF21 binding to the FGFR1/β–klotho complex activated the MAPK and then the mTOR/ribosomal protein S6 kinase (S6K) pathway, which aroused an autocrine feedback loop, increasing the release of FGF21 [45]. Therefore, FGF21 secretion was enhanced 3.0- and 4.4-fold (*p* < 0.05) in basal and NAFLD conditions, respectively. Although PA can activate FGF21, as observed in obese and fasting states [46], FGF21 concentration only increased by 33% (*p* < 0.05), consistently with previous reports [47,48]. It is worth noting that PA might conduce FGF21 resistance due to diminished FGFR/β-KL expression. Therefore, although increasing FGF21 secretion and ERK phosphorylation, PA produces detrimental effects by blocking active FGF21 signaling [49] (Figure 2H). Hence, we observed that the major phytochemicals from the cocoa shell could trigger ERK phosphorylation via FGFR activation and consequently favor the release of FGF21, independently of the presence of PA (Figure 2H). These results suggest the potential involvement of cocoa phytochemicals on hepatic cell signaling and subsequent metabolic events.

### 3.3. Cocoa Shell Phytochemicals, Mainly Flavanols, Preserved Cell Viability and Reduced Inflammation

**Figure 3:**
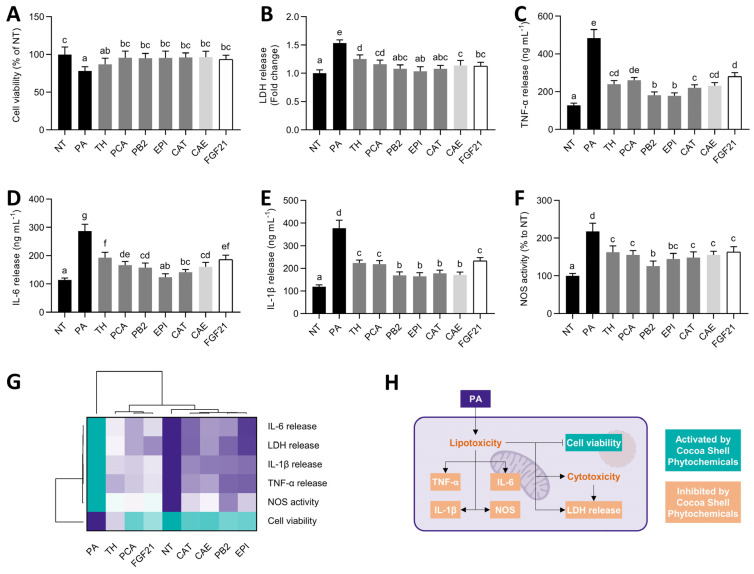
Role of pure phytochemicals from the cocoa shell (50 µmol L^−1^), aqueous extract (CAE, 100 µg mL^−1^), and FGF21 (20 nmol L^−1^), in the presence of palmitic acid (PA, 500 µmol L^−1^), on lipo/cytotoxicity and inflammation markers in HepG2 human hepatocytes. Cocoa shell phytochemicals preserved cell viability (**A**) and diminished lactate dehydrogenase (LDH) release (**B**). PA-triggered TNF-α (**C**), IL-6 (**D**), and IL-1β (**E**) release and nitric oxide synthase (NOS) activity (**F**) were reduced. Hierarchical cluster analysis and heat map (from the lowest (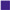) to the highest (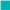) value for each parameter) (**G**) and an integrative diagram illustrating the effects of the phytochemicals from the cocoa shell on lipo/cytotoxicity and inflammation (**H**). The results are expressed as mean ± SD (*n* = 3). Bars with different letters (a–f) significantly (*p* < 0.05) differ according to ANOVA and Tukey’s multiple range test. NT: non-treated cells; TH: theobromine; PCA: protocatechuic acid; PB2: procyanidin B2; EPI: epicatechin; CAT: catechin; FGF21: fibroblast growth factor 21.

Considering the adverse effects of PA on HepG2 cells [50], we aimed to evaluate the co-treatment with the main cocoa shell phytochemicals to evaluate their effects in NAFLD progression in the cell model and ensure no synergistic adverse effects with PA (Figure 3). Upon PA treatment, hepatocytes suffered reductions (22%) in cell viability and an increase in LDH release (54%) (Figure 3A,B), indicating that stimulating HepG2 with PA causes lipo/cytotoxicity. Cocoa shell phytochemicals and the aqueous extract significantly (*p* < 0.05) reversed these effects. These effects were also observed for the FGF21 treatment. Concomitantly, PA triggered an inflammatory response in hepatocytes (Figure 3C–E). The release of TNF-α, IL-6, and IL-1β was increased (3.8-, 2.5-, and 3.2-fold, respectively). The studied pure phytochemicals from the cocoa shell, CAE, and FGF21 diminished TNF-α release by 42–63%. Similarly, IL-6 and IL-1β secretion was reduced by 33–57% and 38–56%, respectively. Flavanols, PB2, EPI, and CAT exhibited significantly (*p* < 0.05) higher effects than the other treatments. Increased NOS activity (2.2-fold) was observed in PA-treated hepatocytes, but the phytochemicals from the cocoa shell, mainly PB2, counteracted these effects (25–42%, *p* < 0.05) (Figure 5F). According to multivariate analyses (Figure 3G), all treatments abrogated PA’s lipotoxic and inflammatory effects. Flavanols (PB2, EPI, and CAT) were the most effective compounds in CAE, whereas TH and PCA showed similar effects as FGF21. Since PA can induce cell damage and apoptosis [51], treatments for preventing these effects can be considered for avoiding lipotoxicity-driven hepatosteatosis and NAFLD progression to non-alcoholic steatohepatitis (NASH) [52] (Figure 2H). In conjunction with hepatic steatosis, an inflammatory response occurs in hepatocytes in NAFLD conditions needing to be repressed [53]. Cocoa phytochemicals, mainly the flavanols, PB2, EPI, and CAT, demonstrated their ability to reduce cytokine release and NOS activity (Figure 2H). Previous studies have demonstrated PA activation of nuclear factor kappa-light-chain-enhancer of activated B cells (NF-κB) and its suppression by food bioactive compounds [54]. In addition, FGF21 can also reduce TNF-α expression in PA-treated HepG2 cells [43]. Thence, the cocoa shell could inhibit the evolution of NAFLD, alleviating the effects of saturated fatty acids on the liver.

### 3.4. Cocoa Shell Phytochemicals, Mainly Protocatechuic Acid, Diminished Oxidative Stress via Regulation of Antioxidative Systems

**Figure 4:**
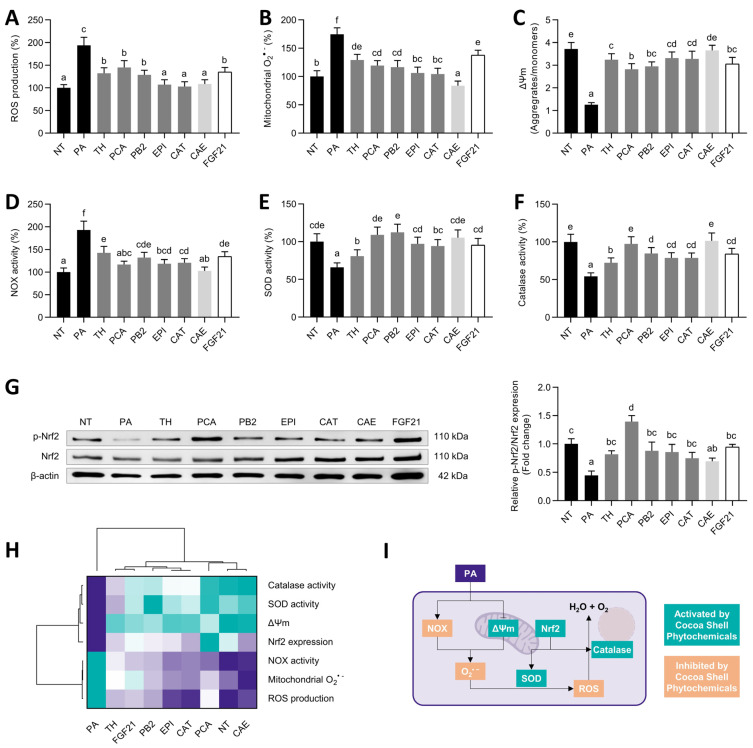
Protective effects of pure phytochemicals from the cocoa shell (50 µmol L^−1^), aqueous extract (CAE, 100 µg mL^−1^), and FGF21 (20 nmol L^−1^), in the presence of palmitic acid (PA, 500 µmol L^−1^) against oxidative stress in HepG2 human hepatocytes. Phytochemicals from the cocoa shell reduced the production of reactive oxygen species (ROS) (**A**) and mitochondrial O_2_^•−^ (**B**) while maintaining the mitochondrial membrane potential (ΔΨm) (**C**) in PA-treated hepatocytes. The enzymatic activity of NADPH oxidase (NOX) (**D**), superoxide dismutase (SOD) (**E**), and catalase (**F**) were regulated, thereby diminishing oxidative stress via the activation of the Nrf2 pathway by stimulating its protein expression and phosphorylation (**G**). Hierarchical cluster analysis and heat map (from the lowest (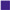) to the highest (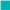) value for each parameter) (**H**) and an integrative diagram illustrating the effects of the phytochemicals from the cocoa shell on oxidative stress (**I**). The results are expressed as mean ± SD (*n* = 3). Bars with different letters (a–f) significantly (*p* < 0.05) differ according to ANOVA and Tukey’s test. NT: non-treated cells; TH: theobromine; PCA: protocatechuic acid; PB2: procyanidin B2; EPI: epicatechin; CAT: catechin; FGF21: fibroblast growth factor 21.

Oxidative stress is one of the second hits of NAFLD. Once hepatocytes are challenged to PA, oxidative stress markers denote cell damage [55]. ROS can be produced in cells by PA-induced enhanced NAPDH oxidase activity or by mitochondria dysfunction. We observed an exacerbated ROS (1.9-fold) and mitochondrial O_2_^•−^ (1.7-fold) production (Figure 4A,B) that was mitigated by the cocoa shell phytochemicals and FGF21 (30–47% and 21–42%, respectively). These effects were inversely associated with the loss of mitochondrial membrane potential, reduced by 67%, and significantly (*p* < 0.05) augmented by the cocoa shell pure compounds and FGF21 (63–84%) and restored by CAE (Figure 4C). Likewise, PA elicited NADPH oxidase activity (1.9-fold), reduced by 26–47% by cocoa shell phytochemicals and FGF21 (Figure 4D). Lastly, cocoa shell phytochemicals increased cellular antioxidant systems activity. PA diminished SOD (34%) and catalase (46%) activities; however, these activities were enhanced (1.2- to 1.7- and 1.3- to 1.9-fold, respectively) by the phytochemicals from the cocoa shell, and by FGF21 (Figure 4E,F).

We observed an increase in the phosphorylation (and therefore activation) of nuclear factor (erythroid-derived 2)-like 2 (Nrf2) (1.6- to 3.1-fold, *p* < 0.05) (Figure 4G). The hierarchical analysis (Figure 4H) demonstrated that CAE completely counteracted PA-derived oxidative stress. Mimicking NAFLD conditions, PA can drive oxidative stress to hepatocytes via mitochondrial dysfunction (see Section 3.5) or by activating NADPH oxidases (Figure 4I) [55]. Here we observed both increased NADPH oxidase activity and ROS and mitochondrial O_2_^•−^ production, reversed by the treatments with the phytochemicals from the cocoa shell. These biomolecules could be working directly, scavenging the ROS overload, or indirectly, stimulating SOD and catalase activity. In turn, FGF21 has been shown to promote this antioxidant enzyme activity under inflammatory conditions [56], leading to the subsequent conversion of O_2_^•−^ into H_2_O_2_, and the latter into water and oxygen. FGF21 signaling activation leads to the expression of antioxidant enzymes via ERK/Nrf2 pathway regulation [57]. PCA, EPI, and CAT, among the other phytochemicals, can activate PA-dysregulated ERK/Nrf2 pathways and elicit the expression and activity of antioxidant enzymes, reducing ROS [58,59,60]. Some studies are currently encouraging the use of vitamin E and certain phenolic compounds to prevent NAFLD oxidative stress [55]. Therefore, cocoa phytochemicals and FGF21 should be considered active therapeutic agents, lessening the impact of saturated fatty acids in the liver, and preventing hepatic oxidative stress.

### 3.5. Protocatechuic Acid and Catechin, among Other Cocoa Shell Phytochemicals, Mimicked FGF21 Protecting Hepatocytes from Mitochondrial Dysfunction

**Figure 5:**
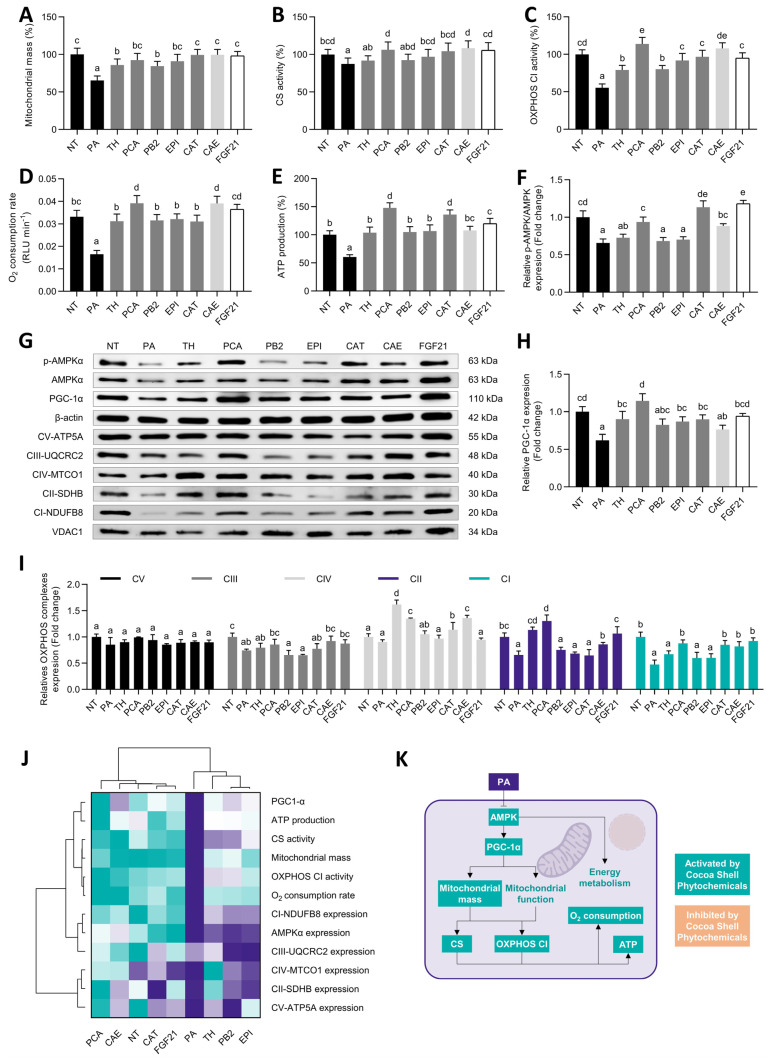
Regulative effects of pure phytochemicals from the cocoa shell (50 µmol L^−1^), aqueous extract (CAE, 100 µg mL^−1^), and FGF21 (20 nmol L^−1^), in the presence of palmitic acid (500 µmol L^−1^), on the mitochondrial function of HepG2 human hepatocytes. Cocoa shell phytochemicals attenuated the loss of mitochondrial mass (**A**) and mitochondrial function as measured by citrate synthase (CS) activity (**B**), oxidative phosphorylation (OXPHOS) complex I (CI) activity (**C**), O_2_ consumption rate (**D**), and ATP production (**E**). HepG2 cells exhibited modulated AMPK expression and phosphorylation (**F**), as shown in Western blot results (**G**). PGC-1α (**H**), and mitochondrial OXPHOS complexes (**I**) protein expression were also regulated. Hierarchical cluster analysis and heat map (from the lowest (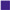) to the highest (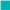) value for each parameter) (**J**) and an integrative diagram illustrating the effects of the phytochemicals from the cocoa shell on mitochondrial function (**K**). The results are expressed as mean ± SD (*n* = 3). Bars with different letters (a–e) significantly (*p* < 0.05) differ according to ANOVA and Tukey’s multiple range test. NT: non-treated cells; PA: palmitic acid; TH: theobromine; PCA: protocatechuic acid; PB2: procyanidin B2; EPI: epicatechin; CAT: catechin; FGF21: fibroblast growth factor 21.

As previously noted, PA can cause oxidative stress in hepatocytes by dysregulating mitochondrial function. Mitochondrial dysfunction, in turn, engenders more oxidative stress and inflammation, creating a vicious cycle [61]. PA-treated hepatocytes showed reduced mitochondrial mass (35%), recovered by CAE, the pure phytochemicals, and FGF21 (55–99%, *p* < 0.05) (Figure 5A). Comparably to previous reports [50], CS activity was just slightly (13%, *p* < 0.05) reduced by PA stimulation; the phytochemicals from the cocoa shell fully reverted this effect (Figure 5B). In contrast, the activity of the CI from the OXPHOS system suffered a detrimental reduction upon the PA challenge (45%, *p* < 0.05) (Figure 5C). The cocoa shell phytochemicals increased OXPHOS CI activity (from 1.4- to 2.1-fold, *p* < 0.05), remarking the PCA-preventive effect. The oxygen consumption rate diminishment caused by PA (50%) was prevented by all phytochemicals and FGF21 and even increased by PCA and CAE (18%) (Figure 5D). As a result of the mitochondrial oxidative phosphorylation, ATP was generated. Here we observed reduced ATP production (39%), neutralized by the cocoa shell phytochemicals, which even increased it (PCA by 48% and CAT by 36%, *p* < 0.05) (Figure 5E). The phosphorylation of AMP-activated protein kinase (AMPK)α (Figure 5F) and the expression of peroxisome proliferator-activated receptor-gamma coactivator (PGC)1-α (Figure 5G,H) were enhanced (4–79% and 23–84%, respectively) in comparison with PA-challenged cells. The protein expression of the different OXPHOS complexes was also regulated (Figure 5I). OXPHOS CV expression did not vary among treatments; CIII expression was slightly increased by PCA, CAE, and FGF21 (15–24%, *p* < 0.05). OXPHOS CIV expression was enhanced around 5–79%, and CII around 4–99% (*p* < 0.05). Finally, the protein expression of the OXPHOS CI was augmented by 1.4- to 1.9-fold (*p* < 0.05) in comparison with PA-stimulated cells, with 53% reduced expression. Multivariate analysis showed similar behavior between FGF21 and CAE, highlighting the ability of PCA and CAT to enhance mitochondrial function (Figure 5J).

NAFLD progression is accompanied by mitochondrial dysfunction. This condition is characterized by reduced OXPHOS activity, O_2_ consumption, and ATP production (Figure 5K), but also by disrupted TCA cycle and β-oxidation (see Section 3.6) [62]. The downregulation of OXPHOS subunits has been associated with NAFLD and has been proven in vitro in PA-stimulated HepG2 cells [50]. As the authors stated, these effects might be associated with exacerbated oxidative stress in the liver, potentially due to NOX increased activity, via AMPK inhibition [50].

Results proved the ability of the cocoa shell phytochemicals, mainly PCA and CAT, to restore mitochondrial content, OXPHOS C1 activity, O_2_ consumption, and ATP production in PA-treated hepatocytes via AMPK/PGC-1α pathways. Our group previously observed similar effects by treating 3T3-L1 adipocytes with PCA, indicating that these events were partially coordinated by AMPK pathway activation [63]. Similarly, CAT has shown considerable effects on reducing PA-induced NAFLD and mitochondrial dysfunction in hepatocytes, avoiding the loss of mitochondrial mass and membrane potential, and stimulating ATP production via the up-phosphorylation of AMPKα^T172^ [64,65]. FGF21 can activate AMPK, sirtuin 1 (SIRT1), and PCG-1α pathways, stimulating mitochondrial respiration [66]. Recently, cocoa consumption has been associated with mitigating NAFLD and mitochondrial preservation in vivo [67]. Another study demonstrated the role of astaxanthin on attenuating hepatic damage and mitochondrial dysfunction in NAFLD in vitro and animal models by up-regulating FGF21/PGC-1α signaling [68]. By silencing FGF21 expression, they demonstrated that the carotenoid preventive properties required FGF21. Correspondingly, FGF21 administration can prevent NAFLD-derived mitochondrial dysfunction in mice [69]. Thus, both PCA and CAT seem to be the main contributors of CAE potential to protect mitochondrial function and exhibit similar effects to FGF21. Therefore, the phytochemicals from the cocoa shell should be considered active compounds in preventing NAFLD and its progression by minimizing the dysregulation of mitochondrial oxidative respiration.

### 3.6. Cocoa Shell Phytochemicals, Primarily Protocatechuic Acid, Reduced the Hepatic Lipid Load by Diminishing De Novo Fatty Acid Synthesis and Stimulation Fatty Acid Oxidation

**Figure 6:**
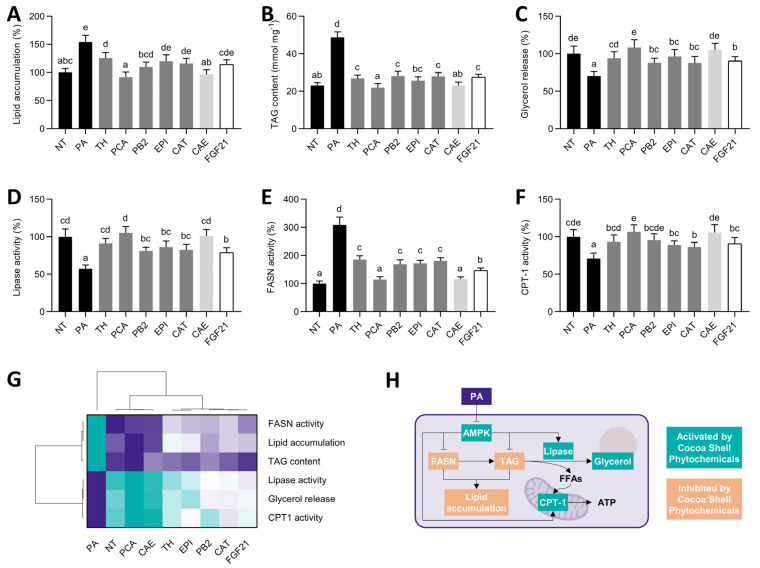
Modulatory effects of pure phytochemicals from the cocoa shell (50 µmol L^−1^), aqueous extract (CAE, 100 µg mL^−1^), and FGF21 (20 nmol L^−1^), in the presence of palmitic acid (500 µmol L^−1^), on lipid metabolism in HepG2 human hepatocytes. Palmitic acid-treated hepatocytes showed reduced lipid accumulation (**A**), diminished intracellular TAG content (**B**), and increased lipolysis measured by glycerol release (**C**) and lipases activity (**D**). The enzymatic activity of fatty acid synthase (FASN) (**E**) and carnitine palmitoyltransferase 1 (CPT-1) (**F**) was regulated by phytochemicals from the cocoa shell. Hierarchical cluster analysis and heat map (from the lowest (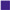) to the highest (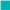) value for each parameter) (**G**) and an integrative diagram illustrating the effects of phytochemicals from the cocoa shell on lipid metabolism (**H**). The results are expressed as mean ± SD (*n* = 3). Bars with different letters (a–e) significantly (*p* < 0.05) differ according to ANOVA and Tukey’s multiple range test. NT: non-treated cells; PA: palmitic acid; TH: theobromine; PCA: protocatechuic acid; PB2: procyanidin B2; EPI: epicatechin; CAT: catechin; FGF21: fibroblast growth factor 21; FFAs: free fatty acids.

The main characteristic of NAFLD is the accumulation of lipids in the liver [70]. Increased lipid accumulation (1.5-fold, *p* < 0.05) was observed in PA-treated hepatocytes (Figure 6A). All treatments significantly reduced the neutral lipid content (53–115%, *p* < 0.05). A similar response was observed for the intracellular TAG content (Figure 6B). TAGs were reduced from 1.7- to 2.2-fold (*p* < 0.05) in comparison with PA-stimulated cells. Regarding lipolysis, the cocoa shell phytochemicals reverted the diminished glycerol release (59–127%) and lipase activity (50–103%) (Figure 6C,D). In addition to investigating free fatty acids mobilization, we analyzed fatty acid synthesis and β-oxidation by evaluating the activity of the key enzymes regulating such processes (FASN and CPT-1) (Figure 6E,F). The phytochemicals from the cocoa shell reduced (1.7- to 2.7-fold, *p* < 0.05) the augmented FASN activity, denoting a down-regulation of de novo fatty acid synthesis. Likewise, the cocoa shell pure compounds, CAE and FGF21, evoked the PA-suppressed CPT-1 activity (1.2- to 1.5-fold, *p* < 0.05). Thus, hierarchical cluster analysis (Figure 6G) demonstrated that PCA was the main compound within CAE’s main phytochemicals, regulating lipid metabolism and dampening PA-derived adverse effects.

Hepatic fat accumulation is caused by an imbalance between lipid uptake and lipid clearance, controlled by four main pathways: absorption of circulating lipids, de novo fatty acid synthesis, fatty acid oxidation, and lipid exportation [70]. PA triggers de novo lipogenesis by reducing AMPK activity. As previously reported, FGF21 down-regulates FASN expression and promotes CPT-1 activity, reducing lipid accumulation and increasing fatty acid oxidation [43]. Consistently, the major studied phytochemicals from the cocoa shell have been demonstrated to regulate these pathways (Figure 6H) and therefore could be modulating them via FGF21 signaling activation. PCA has been recognized as a lipid metabolism regulator, lowering FASN expression and activity in the liver of mice fed a high-fat diet [71], and stimulating CPT-1 expression through the mitochondrial deacetylase SIRT3 pathway [72]. Hence, the phytochemicals found in CAE, primarily PCA, can act as modulators of the key pathways involved in hepatic fat accumulation and consequently prevent NAFLD.

### 3.7. Protocatechuic Acid and Epicatechin, among Cocoa Shell Phytochemicals, Regulated Glucose Metabolism in Palmitic Acid-Challenged Hepatocytes

**Figure 7:**
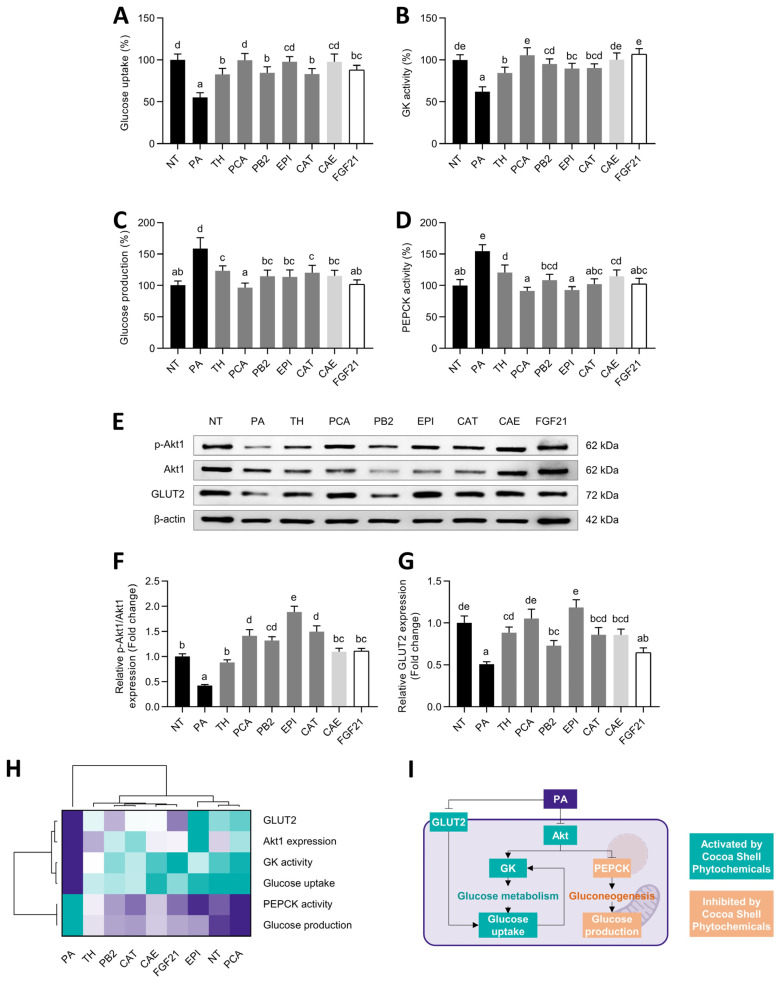
Regulatory effects of pure phytochemicals from the cocoa shell (50 µmol L^−1^), aqueous extract (CAE, 100 µg mL^−1^), and FGF21 (20 nmol L^−1^), in the presence of palmitic acid (500 µmol L^−1^), on glucose metabolism in HepG2 human hepatocytes. Hepatocytes exhibited a modulation on glucose uptake (**A**), glucokinase (GK) activity (**B**), glucose production (**C**), and phosphoenolpyruvate carboxykinase (PEPCK) activity (**D**). Phytochemicals from the cocoa shell regulated the protein expression (**E**) of Akt1 (**F**) and GLUT2 (**G**). Hierarchical cluster analysis and heat map (from the lowest (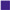) to the highest (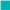) value for each parameter) (**H**) and an integrative diagram illustrating the effects of the phytochemicals from the cocoa shell on glucose metabolism (**I**). The results are expressed as mean ± SD (*n* = 3). Bars with different letters (a–e) significantly (*p* < 0.05) differ according to ANOVA and Tukey’s multiple range test. NT: non-treated cells; PA: palmitic acid; TH: theobromine; PCA: protocatechuic acid; PB2: procyanidin B2; EPI: epicatechin; CAT: catechin; FGF21: fibroblast growth factor 21.

Beyond eliciting lipid metabolism disorders, NAFLD is characterized by hepatic insulin resistance and glucose homeostasis dysregulation [73]. Using PA-treated HepG2 cells as a NAFLD model, we observed a reduced glucose uptake (Figure 7A), which was repressed from 1.5- to 1.8-fold by the cocoa shell phytochemicals and FGF21.

Similarly, glucokinase activity was recovered after PA stimulation and increased 1.4- to 1.7-fold (*p* < 0.05) (Figure 7B). Conversely, the gluconeogenic production of glucose was reduced (60–106%) (Figure 7C), probably due to the suppression of PEPCK activity (1.2- to 1.7-fold, *p* < 0.05) (Figure 7D), a key catalyst in the gluconeogenic process. Akt1 phosphorylation was enhance by 2.1- to 4.5-fold (Figure 7E,F), whereas the glucose transporter 2 (GLUT-2) expression increased by 1.3- to 2.3-fold (*p* < 0.05) (Figure 7G). Multivariate analysis indicated that the cocoa shell phytochemicals, primarily PCA and EPI, preserved glucose metabolism as the NT control (Figure 7H).

Hepatocytes obtain glucose from the bloodstream via GLUT-2. GK phosphorylates glucose to produce glucose 6-phosphate, lowering intracellular glucose concentrations and boosting glucose absorption (Figure 7I) [74]. Thus, here we observed how the cocoa shell phytochemicals, mainly PCA and EPI, promoted glucose uptake and GK activity. EPI and a cocoa phenolic extract (CPE) have been shown to elicit GLUT-2-dependent glucose uptake via AKT and AMPK signaling pathways activation [75]. Comparably, FGF21 prompts GLUT-2 facilitated glucose uptake and GK expression in vivo [76]. On the contrary, gluconeogenesis is regulated by the availability of gluconeogenic substrates as well as the expression/activation of gluconeogenic enzymes (PEPCK and glucose-6-phosphatase) that govern key gluconeogenesis events (Figure 7I) [74]. PCA exhibits hepatoprotective effects in vivo, enhancing PEPCK expression in dexamethasone-treated animals [77]. EPI and CPE also exhibited the potential to reduce glucose production via PEPCK depletion [75]. FGF21 also mediates PEPCK and glucose production diminishments [78]. Liver lipid accumulation contributes to hepatic insulin resistance, which is a risk factor for NAFLD development, creating a vicious cycle. Consequently, insulin resistance blocks glucose uptake and perturbs the insulin-mediated suppression of gluconeogenesis [74]. Then, reducing gluconeogenesis and stimulating glucose metabolism may contribute to reducing glucose blood levels, and further prevent insulin resistance and diabetes [79]. Accordingly, the cocoa shell phytochemicals may modulate glucose metabolism and prevent NAFLD avoiding hyperglycemic states and developing systemic insulin resistance. Further molecular mechanisms will be explained in Section 3.8.

### 3.8. Cocoa Shell Phytochemicals Differentially Modulate Protein Phosphorylation Thereby Regulating mTOR, AKT, and ERK Signaling Pathways

Lipid and glucose metabolism and mitochondrial function were mainly regulated by CAE, PCA, and FGF21, as supported by hierarchical cluster analysis, which demonstrated non-significant (*p* > 0.05) differences among the three groups. The underlying molecular mechanisms governing these NAFLD-preventive effects were partially unveiled in previous sections. Nonetheless, protein phosphorylation patterns in proteins from multiple crucial pathways may also have a causative role on CAE’s effects. The results exhibited the differential phosphorylation pattern of multiple proteins between PA-challenged cells and CAE-treated hepatocytes (Table 1); 22 out of 27 proteins were significantly up-phosphorylated. Among all the proteins, it is noteworthy to highlight the fold change of INSR^Y1189^ (2.87, *p* = 0.009) and PTEN^S370^ (4.65, *p* = 0.008), phosphorylation that activates and deactivates each of the proteins, resulting in the activation of insulin signaling [80]. AMPKα^T172^ increase (2.38, *p* = 0.009) indicates that CAE phytochemicals reverted PA-triggered AMPK signaling inhibition, prompting energy metabolism regulation [81].

**Figure 8:**
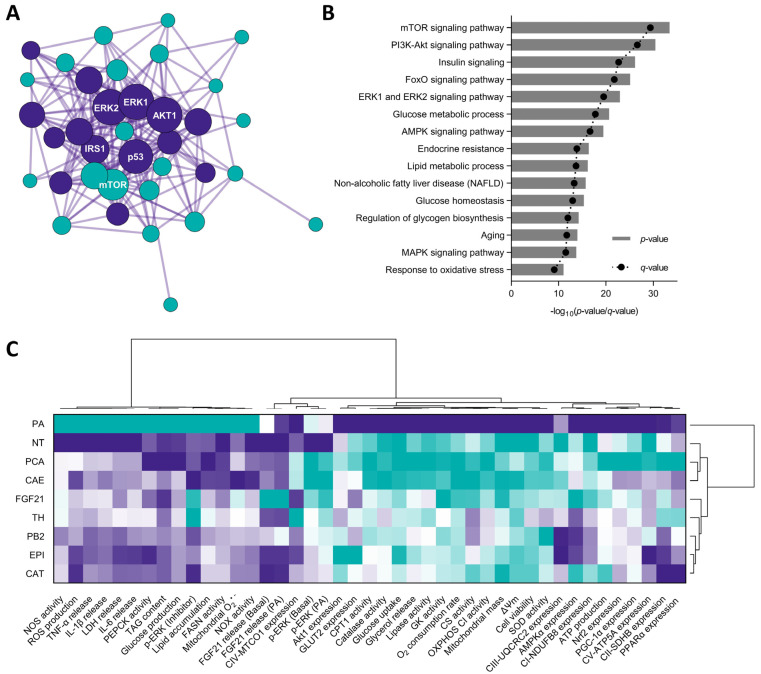
Cocoa shell phytochemicals differentially modulated the phosphorylation of proteins associated with cell signaling pathways. Protein–protein interaction networks built with Metascape from the differentially phosphorylated protein in hepatocytes by the co-treatment of HepG2 cells with palmitic acid (500 µmol L^−1^) and the cocoa shell aqueous extract (CAE, 100 µg mL^−1^) (using cells only treated with palmitic acid as a control) (**A**) and KEGG pathways associated with the differentially phosphorylated proteins (**B**). Hierarchical cluster analysis and heat map (from the lowest (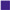) to the highest (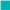) value for each parameter) classifying all the treatments studies according to the effects observed (**C**).

As previously observed, CAE increased ERK1/2 phosphorylation (2.99, *p* = 0.006) and activated the mTOR/S6K pathway by phosphorylating mTOR^T2448^ (2.23, *p* = 0.038) and rpS6^S235/6^ (2.55, *p* = 0.014). Likewise, the increase in the phosphorylation of BAD^S112^ (3.65, *p* = 0.008), GSK3α^S21^ (2.85, *p* = 0.008), RSK1^S380^ (2.45, *p* = 0.008), and RSK2^S368^ (1.96, *p* = 0.009) is remarkable since it indicates a blockade on cell apoptosis and stimulation of cell proliferation. The bioinformatic analysis of differentially expressed/phosphorylated proteins indicated that mTOR, AKT, insulin, FoxO, and ERK1/2 were the top five modulated signaling pathways (Figure 8A,B).

**Figure 9:**
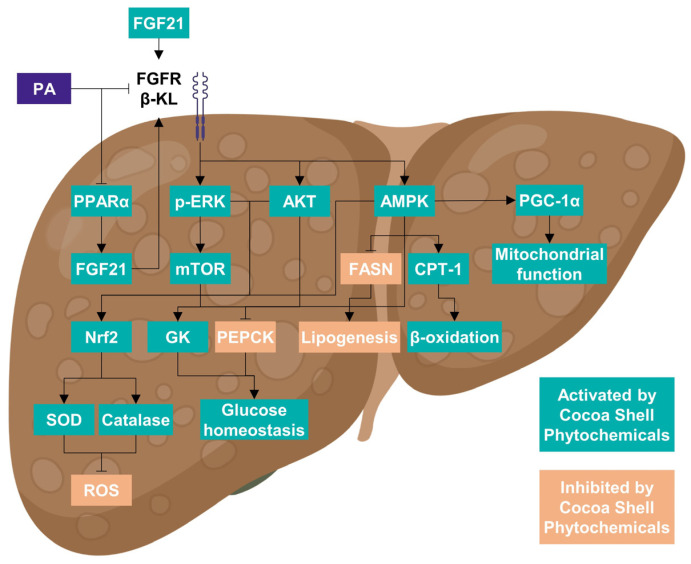
Illustrative diagram of the main events occurring in hepatocytes under non-alcoholic fatty liver disease (NALFD) conditions. The activation of FGF21 signaling by the phytochemicals from the cocoa shell is conducive to the regulation of lipid and glucose metabolism, via ERK, mTOR, AKT, and AMPK pathways. Harmoniously, these metabolic events lead to antioxidant enzymes stimulation and ROS reduction, and to the enhancement of mitochondrial bioenergetic functions. The blockade on liver metabolism generated by saturated fatty acids, here palmitic acid, is repressed, avoiding the development of NAFLD.

PA-challenged HepG2 cells have a less functional phenotype because of the modulation of cell metabolic signals. The stimulation of Nrf2-mediated antioxidative cellular defenses is linked to the activation of the ERK and Akt pathways [82,83]. ERK and Akt pathways have also been linked to hepatocyte mitochondrial function loss [84,85]. As a result, the phytochemicals from the cocoa shell may be counteracting PA-negative effects stimulating antioxidant enzymes (SOD and catalase) and a proper mitochondrial function (OXPHOS activity and ATP production). Likewise, the CAE phytochemicals might stimulate the ERK-activated mTOR/SK6 pathway, essential for reducing gluconeogenesis and developing hyperglycemia and insulin resistance [86]. The activation of the AMPK pathway suppresses de novo lipogenesis in the liver and increases fatty acid oxidation [87], which agrees with our results. AMPK phosphorylates and inactivates acetyl-CoA carboxylase (ACC), resulting in a decrease in malonyl-CoA, an inhibitor of mitochondrial fatty acid oxidation rate-limiting enzyme CPT-1 [87]. Correspondingly, FGF21 activates ERK, Akt, mTOR, and AMPK pathways [88]. Thence, CAE phytochemicals, mainly PCA, could be mimicking FGF21 action, consequently protecting liver cells from the free fatty acid overburden. As observed in multivariate analysis (Figure 8C), CAE was able to revert PA-derived NAFLD biomarkers in hepatocytes, which would mainly derive from the presence of PCA since their total effects were significantly comparable. Theobromine and flavanols may also significantly (*p* < 0.05) protect hepatocytes imitating FGF21 (all treatments significantly differed from PA-treated cells). Previously, PCA was proven to imitate insulin and modulate downstream signaling pathways [89]. Figure 9 summarizes the main events elicited by PA in hepatocytes mimicking NAFLD conditions and the effects of the cocoa shell phytochemicals alleviating adverse effects by regulating energy metabolism and mitochondrial function.

Despite the potential in vitro biological activity of the cocoa shell phytochemicals, methylxanthines and phenolic compounds, limited bioavailability constrains their effectiveness [90]. There is only a partial absorption of these phytochemicals in the digestive system. Following metabolism by the microbiota or the liver, phytochemicals’ effects might be somewhat changed [91]. Therefore, future research should be focused on investigating the effects of gastrointestinal digestion and metabolism on the bioactivity of the cocoa shell and exploring the impact of microbiota on the cocoa shell health-promoting effects. Then, cell and animal models could be used to validate the cocoa shell phytochemicals’ bioactivity and uncover their molecular mechanisms of action. Our group recently validated the cocoa shell as an antioxidant food ingredient safe for human consumption [92]. However, future animal and clinical investigations will be necessary to confirm the positive effects observed in vitro and determine the absorption and metabolism of the cocoa shell phytochemicals.

## 4. Conclusions

The revalorization of cocoa by-products as a source of active compounds is suggested as a strategy for preventing NAFLD. This study describes the evaluation of the phytochemicals from the cocoa shell in the activation of FGF21 signaling, the inhibition of oxidative stress and mitochondrial dysfunction, and the modulation of lipid and glucose metabolism using HepG2 human hepatocytes. We present new knowledge on the mechanisms of action of the phytochemicals from the cocoa shell in hepatocytes under NAFLD conditions. Together, our results demonstrate that the main phytochemicals from cocoa by-products, especially protocatechuic acid, can trigger FGF21 signaling and attenuate inflammation, oxidative stress, lipid accumulation, and glucose intolerance in HepG2 hepatocytes. Considering the higher concentration of theobromine and protocatechuic acid in the cocoa shell aqueous extract (CAE), it is expected that they will be the primary actors in the effects observed. Further studies are needed to conclude on the specific contribution of each CAE component at the concentrations present in the cocoa-by products, their absorption and metabolism. In conclusion, the results proved, for the first time, the potential positive effects of the phytochemicals from cocoa shell in preventing NAFLD by activating FGF21 signaling, oxidative stress, and mitochondrial dysfunction, and alleviating lipid accumulation and insulin resistance.

## Figures and Tables

**Table 1:** Differential phosphorylation ratio of twenty-seven proteins related to the insulin, AKT, mTOR, FoxO, AMPK, and MAPK pathways in HepG2 cells in response to the treatment with palmitic acid (PA, 500 µmol L^−1^) and the co-treatment with PA and the phytochemical-rich aqueous extract from the cocoa shell (CAE, 100 µg mL^−1^).

Target Protein	Phosphosite	Effect of Phosphorylation	Relative Phosphorylation		Fold Change	
			PA	CAE	CAE/PA	*p*-Value
*Insulin signaling*						
IGF1R	Y1165	Induces activity	0.23 ± 0.02	0.45 ± 0.04	1.99 ± 0.22	0.033 *
INSR	Y1189	Induces activity	0.68 ± 0.05	1.96 ± 0.11	2.87 ± 0.21	0.009 **
IRS-1	S318	Inhibits molecular association	0.06 ± 0.01	0.26 ± 0.03	4.28 ± 0.48	0.020 *
SHC-1	Y427	Induces activity	0.18 ± 0.02	0.32 ± 0.05	1.77 ± 0.35	0.117
SHIP-1	Y1020	Induces activity	0.13 ± 0.01	0.30 ± 0.05	2.21 ± 0.39	0.060
SHP-2	T542	Induces molecular association	0.34 ± 0.03	0.56 ± 0.04	1.65 ± 0.21	0.081
*PI3K-AKT-PKB signaling*						
AKT	S473	Induces activity	0.57 ± 0.04	1.54 ± 0.09	2.71 ± 0.21	0.011 *
BAD	S112	Inhibits molecular association	0.29 ± 0.02	1.08 ± 0.11	3.65 ± 0.32	0.008 **
GSK3α	S21	Inhibits activity	0.46 ± 0.03	1.31 ± 0.08	2.85 ± 0.20	0.008 **
GSK3β	S9	Inhibits activity	0.67 ± 0.02	1.44 ± 0.12	2.15 ± 0.17	0.012 *
PDK1	S241	Induces activity	0.63 ± 0.02	1.24 ± 0.08	1.97 ± 0.14	0.013 *
PTEN	S370	Inhibits activity	0.40 ± 0.05	1.85 ± 0.08	4.65 ± 0.39	0.008 **
*mTOR*/*S6K signaling*						
4E-BP1	T36	Inhibits activity	0.95 ± 0.03	1.77 ± 0.39	1.86 ± 0.35	0.075
EIF4E	S209	Inhibits molecular interaction	0.10 ± 0.01	0.22 ± 0.01	2.12 ± 0.24	0.038 *
mTOR	T2448	Induces activity	0.73 ± 0.07	1.64 ± 0.17	2.23 ± 0.29	0.038 *
p70S6K	T421/S424	Induces activity	0.61 ± 0.02	0.94 ± 0.09	1.54 ± 0.17	0.055
PRAS40	T246	Inhibits activity	0.63 ± 0.04	1.38 ± 0.16	2.19 ± 0.26	0.026 *
rpS6	S235/236	Induces activity	0.87 ± 0.08	2.21 ± 0.06	2.55 ± 0.19	0.014 *
*FoxO signaling*						
FOXO3	S413	Induces activity	0.02 ± 0.00	0.04 ± 0.01	2.12 ± 0.39	0.074
p27	T198	Inhibits molecular interaction	0.38 ± 0.05	1.00 ± 0.10	2.61 ± 0.38	0.038 *
*AMPK signaling*						
AMPKα	T172	Induces activity	0.65 ± 0.03	1.55 ± 0.09	2.38 ± 0.16	0.009 **
LKB1	S428	Induces activity	0.44 ± 0.05	1.00 ± 0.05	2.24 ± 0.25	0.036 *
p53	S15	Induces activity	0.60 ± 0.03	1.70 ± 0.13	2.85 ± 0.20	0.007 *
*MAPK signaling*						
ERK1/2	T202/Y204 Y185/187	Induces activity	0.50 ± 0.02	1.51 ± 0.10	2.99 ± 0.19	0.006 **
Raf-1	S301	Inhibits activity	0.51 ± 0.06	1.48 ± 0.11	2.88 ± 0.33	0.022 *
RSK1	S380	Induces protein degradation	0.49 ± 0.03	1.19 ± 0.05	2.45 ± 0.15	0.008 **
RSK2	S386	Induces activity	0.71 ± 0.03	1.38 ± 0.05	1.96 ± 0.11	0.009 **

Results are reported as mean ± SD (*n* = 3). *p*-values followed by *, **, indicate a statistically significance difference between PA and CAE groups when subjected to the *t*-test (*p* < 0.05, *p* < 0.01, *p* < 0.001, respectively).

## Data Availability

Data are contained within the article.

## Supplementary Materials

The following supporting information can be downloaded at: www.mdpi.com/article/10.3390/antiox11010136/s1, Table S1: Identification parameters and phytochemical composition of cocoa shell aqueous extract (CAE) characterized by UPLC-ESI-MS/MS; Figure S1: Effects of pure phytochemicals from cocoa shell (50 µmol L^−1^), aqueous extract (CAE, 100 µg mL^−1^), and FGF21 (20 nmol L^−1^) on reactive oxygen species (ROS) production (A) and mitochondrial content (B) in HepG2 human hepatocytes.

## References

[1] World Health Organization Noncommunicable Diseases. https://www.who.int/news-room/fact-sheets/detail/noncommunicable-diseases.

[2] Rani V., Deep G., Singh R.K., Palle K., Yadav U.C.S. (2016). Oxidative stress and metabolic disorders: Pathogenesis and therapeutic strategies. Life Sci..

[3] Abd El-Kader S.M., El-Den Ashmawy E.M.S. (2015). Non-alcoholic fatty liver disease: The diagnosis and management. World J. Hepatol..

[4] Younossi Z., Anstee Q.M., Marietti M., Hardy T., Henry L., Eslam M., George J., Bugianesi E. (2018). Global burden of NAFLD and NASH: Trends, predictions, risk factors and prevention. Nat. Rev. Gastroenterol. Hepatol..

[5] Schulze M.B., Martínez-González M.A., Fung T.T., Lichtenstein A.H., Forouhi N.G. (2018). Food based dietary patterns and chronic disease prevention. BMJ.

[6] Tezze C., Romanello V., Sandri M. (2019). FGF21 as Modulator of Metabolism in Health and Disease. Front. Physiol..

[7] So W.Y., Leung P.S. (2016). Fibroblast Growth Factor 21 As an Emerging Therapeutic Target for Type 2 Diabetes Mellitus. Med. Res. Rev..

[8] Fisher M.F., Chui P.C., Antonellis P.J., Bina H.A., Kharitonenkov A., Flier J.S., Maratos-Flier E. (2010). Obesity Is a Fibroblast Growth Factor 21 (FGF21)-Resistant State. Diabetes.

[9] Montagner A., Polizzi A., Fouché E., Ducheix S., Lippi Y., Lasserre F., Barquissau V., Régnier M., Lukowicz C., Benhamed F. (2016). Liver PPARα is crucial for whole-body fatty acid homeostasis and is protective against NAFLD. Gut.

[10] Yie J., Wang W., Deng L., Tam L.-T., Stevens J., Chen M.M., Li Y., Xu J., Lindberg R., Hecht R. (2012). Understanding the Physical Interactions in the FGF21/FGFR/β-Klotho Complex: Structural Requirements and Implications in FGF21 Signaling. Chem. Biol. Drug Des..

[11] Nies V.J.M., Sancar G., Liu W., van Zutphen T., Struik D., Yu R.T., Atkins A.R., Evans R.M., Jonker J.W., Downes M.R. (2016). Fibroblast Growth Factor Signaling in Metabolic Regulation. Front. Endocrinol..

[12] Zarei M., Pizarro-Delgado J., Barroso E., Palomer X., Vázquez-Carrera M. (2020). Targeting FGF21 for the Treatment of Nonalcoholic Steatohepatitis. Trends Pharmacol. Sci..

[13] Panak Balentić J., Ačkar Đ., Jokić S., Jozinović A., Babić J., Miličević B., Šubarić D., Pavlović N. (2018). Cocoa Shell: A By-Product with Great Potential for Wide Application. Molecules.

[14] Rebollo-Hernanz M., Zhang Q., Aguilera Y., Martín-Cabrejas M.A., de Mejia E.G. (2019). Cocoa Shell Aqueous Phenolic Extract Preserves Mitochondrial Function and Insulin Sensitivity by Attenuating Inflammation between Macrophages and Adipocytes In Vitro. Mol. Nutr. Food Res..

[15] Rebollo-Hernanz M., Zhang Q., Aguilera Y., Martín-Cabrejas M.A., de Mejia E.G. (2019). Relationship of the phytochemicals from coffee and cocoa by-products with their potential to modulate biomarkers of metabolic syndrome in vitro. Antioxidants.

[16] Yang W., Chen X., Liu Y., Chen M., Jiang X., Shen T., Li Q., Yang Y., Ling W. (2017). N-3 polyunsaturated fatty acids increase hepatic fibroblast growth factor 21 sensitivity via a PPAR-γ-β-klotho pathway. Mol. Nutr. Food Res..

[17] Ejaz A., Martinez-Guino L., Goldfine A.B., Ribas-Aulinas F., De Nigris V., Ribó S., Gonzalez-Franquesa A., Garcia-Roves P.M., Li E., Dreyfuss J.M. (2016). Dietary Betaine Supplementation Increases Fgf21 Levels to Improve Glucose Homeostasis and Reduce Hepatic Lipid Accumulation in Mice. Diabetes.

[18] Zeng K., Tian L., Patel R., Shao W., Song Z., Liu L., Manuel J., Ma X., McGilvray I., Cummins C.L. (2016). Diet polyphenol curcumin stimulates hepatic Fgf21 production and restores its sensitivity in high fat diet fed male mice. Endocrinology.

[19] Rebollo-Hernanz M., Cañas S., Taladrid D., Bartolomeé B., Aguilera Y., Martin-Cabrejas M.A. (2021). Extraction of phenolic compounds from cocoa shell: Modeling using response surface methodology and artificial neural networks. Sep. Purif. Technol..

[20] Aguilera Y., Rebollo-Hernanz M., Cañas S., Taladrid D., Martín-Cabrejas M.A. (2019). Response surface methodology to optimise the heat-assisted aqueous extraction of phenolic compounds from coffee parchment and their comprehensive analysis. Food Funct..

[21] Vangone A., Schaarschmidt J., Koukos P., Geng C., Citro N., Trellet M.E., Xue L.C., Bonvin A.M.J.J. (2019). Large-scale prediction of binding affinity in protein–small ligand complexes: The PRODIGY-LIG web server. Bioinformatics.

[22] Xue L.C., Rodrigues J.P., Kastritis P.L., Bonvin A.M., Vangone A. (2016). PRODIGY: A web server for predicting the binding affinity of protein–protein complexes. Bioinformatics.

[23] Herrera B., Murillo M.M., Álvarez-Barrientos A., Beltrán J., Fernández M., Fabregat I. (2004). Source of early reactive oxygen species in the apoptosis induced by transforming growth factor-β in fetal rat hepatocytes. Free Radic. Biol. Med..

[24] Zhou J.Y., Prognon P. (2006). Raw material enzymatic activity determination: A specific case for validation and comparison of analytical methods—The example of superoxide dismutase (SOD). J. Pharm. Biomed. Anal..

[25] Wang Z.J., Liang C.L., Li G.M., Yu C.Y., Yin M. (2006). Neuroprotective effects of arachidonic acid against oxidative stress on rat hippocampal slices. Chem. Biol. Interact..

[26] Desquiret-Dumas V., Gueguen N., Leman G., Baron S., Nivet-Antoine V., Chupin S., Chevrollier A., Vessières E., Ayer A., Ferré M. (2013). Resveratrol induces a mitochondrial complex i-dependent increase in nadh oxidation responsible for sirtuin activation in liver cells. J. Biol. Chem..

[27] Rebollo-Hernanz M., Zhang Q., Aguilera Y., Martín-Cabrejas M.A., Gonzalez de Mejia E. (2019). Phenolic compounds from coffee by-products modulate adipogenesis-related inflammation, mitochondrial dysfunction, and insulin resistance in adipocytes, via insulin/PI3K/AKT signaling pathways. Food Chem. Toxicol..

[28] Luna-Vital D., Weiss M., Gonzalez de Mejia E. (2017). Anthocyanins from Purple Corn Ameliorated Tumor Necrosis Factor-α-Induced Inflammation and Insulin Resistance in 3T3-L1 Adipocytes via Activation of Insulin Signaling and Enhanced GLUT4 Translocation. Mol. Nutr. Food Res..

[29] Lin J.J., Liu Y.C., Chang C.J., Pan M.H., Lee M.F., Pan B.S. (2018). Hepatoprotective mechanism of freshwater clam extract alleviates non-alcoholic fatty liver disease: Elucidated: In vitro and in vivo models. Food Funct..

[30] Kololziej M.P., Crilly P.J., Corstorphine C.G., Zammit V.A. (1992). Development and characterization of a polyclonal antibody against rat liver mitochondrial overt carnitine palmitoyltransferase (CPT I). Distinction of CPT I from CPT II and of isoforms of CPT I in different tissues. Biochem. J..

[31] Dhanesha N., Joharapurkar A., Shah G., Dhote V., Kshirsagar S., Bahekar R., Jain M. (2012). Exendin-4 reduces glycemia by increasing liver glucokinase activity: An insulin independent effect. Pharmacol. Rep..

[32] Teng H., Chen L., Song H. (2016). The potential beneficial effects of phenolic compounds isolated from: A. pilosa Ledeb on insulin-resistant hepatic HepG2 cells. Food Funct..

[33] Zhou Y., Zhou B., Pache L., Chang M., Khodabakhshi A.H., Tanaseichuk O., Benner C., Chanda S.K. (2019). Metascape provides a biologist-oriented resource for the analysis of systems-level datasets. Nat. Commun..

[34] Kanehisa M., Furumichi M., Sato Y., Ishiguro-Watanabe M., Tanabe M. (2021). KEGG: Integrating viruses and cellular organisms. Nucleic Acids Res..

[35] Mazzutti S., Rodrigues L.G.G., Mezzomo N., Venturi V., Ferreira S.R.S. (2018). Integrated green-based processes using supercritical CO2 and pressurized ethanol applied to recover antioxidant compouds from cocoa (*Theobroma cacao*) bean hulls. J. Supercrit. Fluids.

[36] Jokić S., Gagić T., Knez Ž., Šubarić D., Škerget M. (2018). Separation of Active Compounds from Food by-Product (Cocoa Shell) Using Subcritical Water Extraction. Molecules.

[37] Mellinas A.C., Jiménez A., Garrigós M.C. (2020). Optimization of microwave-assisted extraction of cocoa bean shell waste and evaluation of its antioxidant, physicochemical and functional properties. LWT.

[38] Pavlović N., Jokić S., Jakovljević M., Blažić M., Molnar M. (2020). Green Extraction Methods for Active Compounds from Food Waste—Cocoa Bean Shell. Foods.

[39] Xu H.Y., Yu L., Chen J.H., Yang L.N., Lin C., Shi X.Q., Qin H. (2020). Sesamol alleviates obesity-related hepatic steatosis via activating hepatic PKA pathway. Nutrients.

[40] Pace E., Jiang Y., Clemens A., Crossman T., Rupasinghe H.P.V. (2018). Impact of thermal degradation of cyanidin-3-O-glucoside of haskap berry on cytotoxicity of hepatocellular carcinoma HepG2 and breast cancer MDA-MB-231 cells. Antioxidants.

[41] Martínez-Garza Ú., Torres-Oteros D., Yarritu-Gallego A., Marrero P.F., Haro D., Relat J. (2019). Fibroblast Growth Factor 21 and the Adaptive Response to Nutritional Challenges. Int. J. Mol. Sci..

[42] Lee S., Choi J., Mohanty J., Sousa L.P., Tome F., Pardon E., Steyaert J., Lemmon M.A., Lax I., Schlessinger J. (2018). Structures of β-klotho reveal a â € zip code’-like mechanism for endocrine FGF signalling. Nature.

[43] Asrih M., Montessuit C., Philippe J., Jornayvaz F.R. (2015). Free Fatty Acids Impair FGF21 Action in HepG2 Cells. Cell. Physiol. Biochem..

[44] Sonoda J., Chen M.Z., Baruch A. (2017). FGF21-receptor agonists: An emerging therapeutic class for obesity-related diseases. Horm. Mol. Biol. Clin. Investig..

[45] Minard A.Y., Tan S.-X., Yang P., Fazakerley D.J., Domanova W., Parker B.L., Humphrey S.J., Jothi R., Stöckli J., James D.E. (2016). mTORC1 Is a Major Regulatory Node in the FGF21 Signaling Network in Adipocytes. Cell Rep..

[46] Ge X., Wang Y., Lam K.S., Xu A. (2012). Metabolic actions of FGF21: Molecular mechanisms and therapeutic implications. Acta Pharm. Sin. B.

[47] Martínez-Fernández L., González-Muniesa P., Sáinz N., Laiglesia L.M., Escoté X., Martínez J.A., Moreno-Aliaga M.J. (2019). Maresin 1 Regulates Hepatic FGF21 in Diet-Induced Obese Mice and in Cultured Hepatocytes. Mol. Nutr. Food Res..

[48] Zang Y., Fan L., Chen J., Huang R., Qin H. (2018). Improvement of Lipid and Glucose Metabolism by Capsiate in Palmitic Acid-Treated HepG2 Cells via Activation of the AMPK/SIRT1 Signaling Pathway. J. Agric. Food Chem..

[49] Dongiovanni P., Crudele A., Panera N., Romito I., Meroni M., De Stefanis C., Palma A., Comparcola D., Fracanzani A.L., Miele L. (2020). β-Klotho gene variation is associated with liver damage in children with NAFLD. J. Hepatol..

[50] García-Ruiz I., Solís-Muñoz P., Fernández-Moreira D., Muñoz-Yagüe T., Solís-Herruzo J.A. (2015). In vitro treatment of HepG2 cells with saturated fatty acids reproduces mitochondrial dysfunction found in nonalcoholic steatohepatitis. Dis. Model. Mech..

[51] Hsu J.-Y., Lin H.-H., Chyau C.-C., Wang Z.-H., Chen J.-H. (2021). Aqueous Extract of Pepino Leaves Ameliorates Palmitic Acid-Induced Hepatocellular Lipotoxicity via Inhibition of Endoplasmic Reticulum Stress and Apoptosis. Antioxidants.

[52] Rada P., González-Rodríguez Á., García-Monzón C., Valverde Á.M. (2020). Understanding lipotoxicity in NAFLD pathogenesis: Is CD36 a key driver?. Cell Death Dis..

[53] Diehl A.M., Day C. (2017). Cause, Pathogenesis, and Treatment of Nonalcoholic Steatohepatitis. N. Engl. J. Med..

[54] Xiao Q., Zhang S., Yang C., Du R., Zhao J., Li J., Xu Y., Qin Y., Gao Y., Huang W. (2019). Ginsenoside Rg1 Ameliorates Palmitic Acid-Induced Hepatic Steatosis and Inflammation in HepG2 Cells via the AMPK/NF- B Pathway. Int. J. Endocrinol..

[55] Delli Bovi A.P., Marciano F., Mandato C., Siano M.A., Savoia M., Vajro P. (2021). Oxidative Stress in Non-alcoholic Fatty Liver Disease. An Updated Mini Review. Front. Med..

[56] Tillman E.J., Rolph T. (2020). FGF21: An Emerging Therapeutic Target for Non-Alcoholic Steatohepatitis and Related Metabolic Diseases. Front. Endocrinol..

[57] Gómez-Sámano M.Á., Grajales-Gómez M., Zuarth-Vázquez J.M., Navarro-Flores M.F., Martínez-Saavedra M., Juárez-León Ó.A., Morales-García M.G., Enríquez-Estrada V.M., Gómez-Pérez F.J., Cuevas-Ramos D. (2017). Fibroblast growth factor 21 and its novel association with oxidative stress. Redox Biol..

[58] Varì R., D’Archivio M., Filesi C., Carotenuto S., Scazzocchio B., Santangelo C., Giovannini C., Masella R. (2011). Protocatechuic acid induces antioxidant/detoxifying enzyme expression through JNK-mediated Nrf2 activation in murine macrophages. J. Nutr. Biochem..

[59] Granado-Serrano A.B., Martín M.A., Haegeman G., Goya L., Bravo L., Ramos S. (2010). Epicatechin induces NF-κB, activator protein-1 (AP-1) and nuclear transcription factor erythroid 2p45-related factor-2 (Nrf2) via phosphatidylinositol-3-kinase/protein kinase B (PI3K/AKT) and extracellular regulated kinase (ERK) signalling in HepG2 cells. Br. J. Nutr..

[60] Talebi M., Talebi M., Farkhondeh T., Mishra G., İlgün S., Samarghandian S. (2021). New insights into the role of the Nrf2 signaling pathway in green tea catechin applications. Phyther. Res..

[61] Li S., Hong M., Tan H.Y., Wang N., Feng Y. (2016). Insights into the Role and Interdependence of Oxidative Stress and Inflammation in Liver Diseases. Oxid. Med. Cell. Longev..

[62] Begriche K., Massart J., Robin M.-A., Bonnet F., Fromenty B. (2013). Mitochondrial adaptations and dysfunctions in nonalcoholic fatty liver disease. Hepatology.

[63] Zhang Q., Gonzalez de Mejia E. (2020). Protocatechuic acid attenuates adipogenesis-induced inflammation and mitochondrial dysfunction in 3T3-L1 adipocytes by regulation of AMPK pathway. J. Funct. Foods.

[64] Rafiei H., Omidian K., Bandy B. (2018). Protection by different classes of dietary polyphenols against palmitic acid-induced steatosis, nitro-oxidative stress and endoplasmic reticulum stress in HepG2 hepatocytes. J. Funct. Foods.

[65] Rafiei H., Omidian K., Bandy B. (2019). Dietary Polyphenols Protect Against Oleic Acid-Induced Steatosis in an in Vitro Model of NAFLD by Modulating Lipid Metabolism and Improving Mitochondrial Function. Nutrients.

[66] Chau M.D.L., Gao J., Yang Q., Wu Z., Gromada J. (2010). Fibroblast growth factor 21 regulates energy metabolism by activating the AMPK–SIRT1–PGC-1α pathway. Proc. Natl. Acad. Sci. USA.

[67] Sun M., Gu Y., Glisan S.L., Lambert J.D. (2021). Dietary cocoa ameliorates non-alcoholic fatty liver disease and increases markers of antioxidant response and mitochondrial biogenesis in high fat-fed mice. J. Nutr. Biochem..

[68] Wu L., Mo W., Feng J., Li J., Yu Q., Li S., Zhang J., Chen K., Ji J., Dai W. (2020). Astaxanthin attenuates hepatic damage and mitochondrial dysfunction in non-alcoholic fatty liver disease by up-regulating the FGF21/PGC-1α pathway. Br. J. Pharmacol..

[69] Lee J.H., Kang Y.E., Chang J.Y., Park K.C., Kim H.-W., Kim J.T., Kim H.J., Yi H.-S., Shong M., Chung H.K. (2016). An engineered FGF21 variant, LY2405319, can prevent non-alcoholic steatohepatitis by enhancing hepatic mitochondrial function. Am. J. Transl. Res..

[70] Ipsen D.H., Lykkesfeldt J., Tveden-Nyborg P. (2018). Molecular mechanisms of hepatic lipid accumulation in non-alcoholic fatty liver disease. Cell. Mol. Life Sci..

[71] Liu W.-H., Lin C.-C., Wang Z.-H., Mong M.-C., Yin M.-C. (2010). Effects of Protocatechuic Acid on Trans Fat Induced Hepatic Steatosis in Mice. J. Agric. Food Chem..

[72] Sun R., Kang X., Zhao Y., Wang Z., Wang R., Fu R., Li Y., Hu Y., Wang Z., Shan W. (2020). Sirtuin 3-mediated deacetylation of acyl-CoA synthetase family member 3 by protocatechuic acid attenuates non-alcoholic fatty liver disease. Br. J. Pharmacol..

[73] Loomba R., Friedman S.L., Shulman G.I. (2021). Mechanisms and disease consequences of nonalcoholic fatty liver disease. Cell.

[74] Rui L. (2014). Energy Metabolism in the Liver. Compr. Physiol..

[75] Cordero-Herrera I., Martín M.A., Bravo L., Goya L., Ramos S. (2013). Cocoa flavonoids improve insulin signalling and modulate glucose production via AKT and AMPK in HepG2 cells. Mol. Nutr. Food Res..

[76] Blanco A.M., Bertucci J.I., Unniappan S. (2020). FGF21 Mimics a Fasting-Induced Metabolic State and Increases Appetite in Zebrafish. Sci. Rep..

[77] El-Sonbaty Y.A., Suddek G.M., Megahed N., Gameil N.M. (2019). Protocatechuic acid exhibits hepatoprotective, vasculoprotective, antioxidant and insulin-like effects in dexamethasone-induced insulin-resistant rats. Biochimie.

[78] Liu J., Yang K., Yang J., Xiao W., Le Y., Yu F., Gu L., Lang S., Tian Q., Jin T. (2019). Liver-derived fibroblast growth factor 21 mediates effects of glucagon-like peptide-1 in attenuating hepatic glucose output. EBioMedicine.

[79] Chao H.-W., Chao S.-W., Lin H., Ku H.-C., Cheng C.-F. (2019). Homeostasis of Glucose and Lipid in Non-Alcoholic Fatty Liver Disease. Int. J. Mol. Sci..

[80] Matsuda S., Kobayashi M., Kitagishi Y. (2013). Roles for PI3K/AKT/PTEN Pathway in Cell Signaling of Nonalcoholic Fatty Liver Disease. ISRN Endocrinol..

[81] Foretz M., Even P.C., Viollet B. (2018). AMPK activation reduces hepatic lipid content by increasing fat oxidation in vivo. Int. J. Mol. Sci..

[82] Lv H., Ren H., Wang L., Chen W., Ci X. (2015). Lico A Enhances Nrf2-Mediated Defense Mechanisms against t -BHP-Induced Oxidative Stress and Cell Death via Akt and ERK Activation in RAW 264.7 Cells. Oxid. Med. Cell. Longev..

[83] Jasek-Gajda E., Jurkowska H., Jasińska M., Lis G.J. (2020). Targeting the MAPK/ERK and PI3K/AKT Signaling Pathways Affects NRF2, Trx and GSH Antioxidant Systems in Leukemia Cells. Antioxidants.

[84] Li Y., Ding H., Liu L., Song Y., Du X., Feng S., Wang X., Li X., Wang Z., Li X. (2020). Non-esterified Fatty Acid Induce Dairy Cow Hepatocytes Apoptosis via the Mitochondria-Mediated ROS-JNK/ERK Signaling Pathway. Front. Cell Dev. Biol..

[85] Dou X., Ding Q., Lai S., Jiang F., Song Q., Zhao X., Fu A., Moustaid-Moussa N., Su D., Li S. (2020). Salidroside alleviates lipotoxicity-induced cell death through inhibition of TLR4/MAPKs pathway, and independently of AMPK and autophagy in AML-12 mouse hepatocytes. J. Funct. Foods.

[86] Hagiwara A., Cornu M., Cybulski N., Polak P., Betz C., Trapani F., Terracciano L., Heim M.H., Rüegg M.A., Hall M.N. (2012). Hepatic mTORC2 activates glycolysis and lipogenesis through Akt, glucokinase, and SREBP1c. Cell Metab..

[87] Smith B.K., Marcinko K., Desjardins E.M., Lally J.S., Ford R.J., Steinberg G.R. (2016). Treatment of nonalcoholic fatty liver disease: Role of AMPK. Am. J. Physiol. Endocrinol. Metab..

[88] Xie T., Leung P.S. (2017). Fibroblast growth factor 21: A regulator of metabolic disease and health span. Am. J. Physiol. Endocrinol. Metab..

[89] Scazzocchio B., Varì R., Filesi C., Del Gaudio I., D’Archivio M., Santangelo C., Iacovelli A., Galvano F., Pluchinotta F.R., Giovannini C. (2015). Protocatechuic acid activates key components of insulin signaling pathway mimicking insulin activity. Mol. Nutr. Food Res..

[90] Kim J., Kim J., Shim J., Lee C.Y., Lee K.W., Lee H.J. (2014). Cocoa Phytochemicals: Recent Advances in Molecular Mechanisms on Health. Crit. Rev. Food Sci. Nutr..

[91] Bohn T., Mcdougall G.J., Alegría A., Alminger M., Arrigoni E., Aura A.M., Brito C., Cilla A., El S.N., Karakaya S. (2015). Mind the gap-deficits in our knowledge of aspects impacting the bioavailability of phytochemicals and their metabolites-a position paper focusing on carotenoids and polyphenols. Mol. Nutr. Food Res..

[92] Rebollo-Hernanz M., Cañas S., Aguilera Y., Benitez V., Gila-Díaz A., Rodriguez-Rodriguez P., Cobeta I.M., de Pablo A.L.L., Gonzalez M.C., Arribas S.M. (2020). Validation of Cocoa Shell as a Novel Antioxidant Dietary Fiber Food Ingredient: Nutritional Value, Functional Properties, and Safety. Curr. Dev. Nutr..

