# Supplementary material for: Phytochemicals from the Cocoa Shell Modulate Mitochondrial Function, Lipid and Glucose Metabolism in Hepatocytes via Activation of FGF21/ERK, AKT, and mTOR Pathways
Source: Antioxidants (Basel). 2022 Jan 8;11(1):136. doi: 10.3390/antiox11010136 (PMC8772970; doi:10.3390/antiox11010136)
Supplement: Supplementary file 1 [file antioxidants-11-00136-s001.zip › antioxidants-1529979-supplementary.pdf]

antioxidants

MDPI

Article

Phytochemicals from the Cocoa Shell Modulate Mitochondrial Function, Lipid and Glucose Metabolism in Hepatocytes via Activation of FGF21/ERK, AKT, and mTOR Pathways

Miguel Rebollo-Hernanz [1,2,3], Yolanda Aguilera [1,2], Maria A. Martin-Cabrejas [1,2] and Elvira Gonzalez de Mejia [3,*]

[1] Department of Agricultural Chemistry and Food Science, Universidad Autónoma de Madrid, 28049 Madrid, Spain; miguel.rebollo@uam.es (M.R.H.); yolanda.aguilera@uam.es (Y.A.); maria.martin@uam.es (M.A.M.C.)
[2] Institute of Food Science Research, CIAL (UAM-CSIC), 28049 Madrid, Spain
[3] Department of Food Science and Human Nutrition, University of Illinois at Urbana-Champaign, Urbana, IL 61801, USA
* Correspondence: edemejia@illinois.edu; Tel.: +1-217-244-3196 (E.G.d.M.)

Abstract: The cocoa shell is a by-product that may be revalorized as a source of bioactive compounds to prevent chronic cardiometabolic diseases. This study aimed to investigate the phytochemicals from cocoa shell as targeted compounds for activating fibroblast growth factor 21 (FGF21) signaling and regulating non-alcoholic fatty liver disease (NAFLD)-related biomarkers linked to oxidative stress, mitochondrial function, and metabolism in hepatocytes. HepG2 cells treated with palmitic acid (PA, 500 μmol L^{-1}) were used in a NAFLD cell model. Phytochemicals from cocoa shell (50 μmol L^{-1}) and an aqueous extract (CAE, 100 μg mL^{-1}) enhanced ERK1/2 phosphorylation (1.7 to 3.3-fold) and FGF21 release (1.4 to 3.4-fold). Mitochondrial function (mitochondrial respiration and ATP production) was protected. Cocoa shell phytochemicals reduced lipid accumulation (53–115%) and fatty acid synthase activity (59–93%) and prompted CPT-1 activity. Glucose uptake and glucokinase activity were enhanced, whereas glucose production and phosphoenolpyruvate carboxykinase activity diminished. The increase in the phosphorylation of the insulin receptor, AKT, AMPKα, mTOR, and ERK1/2 conduced to the regulation of hepatic mitochondrial function and energy metabolism. For the first time, the cocoa shell phytochemicals are proved to modulate FGF21 signaling. Results demonstrate the *in vitro* preventive effect of the phytochemicals from cocoa shell on NAFLD.

Keywords: cocoa shell; cocoa by-products; antioxidants; theobromine; phenolic compounds; phytochemicals; non-alcoholic fatty liver disease; oxidative stress; mitochondrial function; metabolism

Supplementary Table S1. Identification parameters and phytochemical composition of cocoa shell aqueous extract (CAE) characterized by UPLC-ESI-MS/MS. Values are expressed as mean ± SD (n = 3).

Compound	R_t (min)	Mass spectral data		Concentration (μg/g extract)	% of ∑ negative mode	Chemical structure
		[M–H]⁻ (m/z)	MS^2 (m/z)			
Hydroxybenzoic acids						
Gallic acid	1.73	169	125	19.2 ± 0.4	1.1	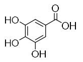
Protocatechuic acid	3.34	153	109	761.5 ± 47.6	45.0	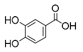
4-hydroxybenzoic acid	4.43	137	93	70.1 ± 9.3	4.1	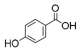
Salicylic acid	8.96	137	93	3.3 ± 0.2	0.2	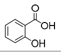
Hydroxycinnamic acids						
Caffeic acid	5.48	179	135	1.9 ± 0.2	0.1	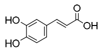
p-coumaric acid	6.81	163	119	4.2 ± 0.6	0.2	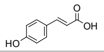
Mandelic acids						
Mandelic acid	4.63	151	107	11.18 ± 1.2	0.7	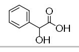
Phenylacetic acids						
3,4-dihydroxyphenylacetic acid	4.18	167	123	25.9 ± 2.4	1.5	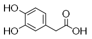
4-hydroxyphenylacetic acid	5.22	151	107	48.5 ± 4.3	2.9	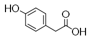
Flavan-3-ols: monomers						
(+)-catechin	5.80	289	245	200.8 ± 16.0	11.9	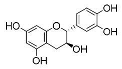
(–)-epicatechin	6.27	289	245	222.1 ± 13.8	13.1	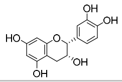
Flavan-3-ols: dimers						
Procyanidin B1	4.90	577	289	83.6 ± 7.8	4.9	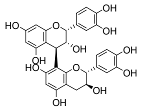
Procyanidin B2	5.93	577	289	219.9 ± 11.4	13.0	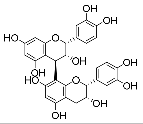
Flavonols						
Quercetin-3-*O*-galactoside	8.34	463	301	9.3 ± 0.4	0.5	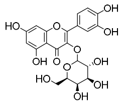
Quercetin-3-*O*-glucoside	8.65	463	301	11.12 ± 0.77	0.7	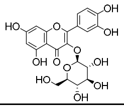

Compound	R_t (min)	Mass spectral data		Concentration (μg/g extract)	% of ∑ positive mode	Chemical structure
		[M–H]⁺ (m/z)	MS^2 (m/z)			
Alkaloids						
Theobromine	2.67	181	138	10035.0 ± 4.5	80.5	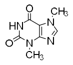
Caffeine	5.46	195	138	2433.5 ± 7.8	19.5	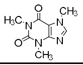

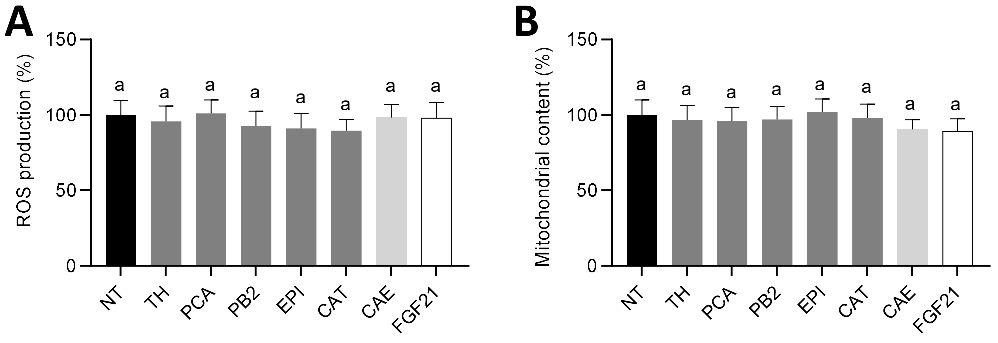

Supplementary Figure S1. Effects of pure phytochemicals from cocoa shell (50 µmol L^{-1}), aqueous extract (CAE, 100 µg mL^{-1}), and FGF21 (20 nmol L^{-1}) on reactive oxygen species (ROS) production (A) and mitochondrial content (B) in HepG2 human hepatocytes. The results are expressed as mean ± SD (n = 3). Bars with different letters significantly ($p < 0.05$) differ according to ANOVA and Tukey's multiple range test. NT: non-treated cells; TH: theobromine; PCA: protocatechuic acid; PB2: procyanidin B2; EPI: epicatechin; CAT: catechin; FGF21: fibroblast growth factor 21.
